# Systematic review and meta-analysis on the use of human platelet lysate for mesenchymal stem cell cultures: comparison with fetal bovine serum and considerations on the production protocol

**DOI:** 10.1186/s13287-022-02815-1

**Published:** 2022-04-04

**Authors:** Silvia Palombella, Carlotta Perucca Orfei, Greta Castellini, Silvia Gianola, Silvia Lopa, Maddalena Mastrogiacomo, Matteo Moretti, Laura de Girolamo

**Affiliations:** 1 Cell and Tissue Engineering Laboratory, IRCCS Istituto Ortopedico Galeazzi, 20161 Milan, Italy; 2 Laboratorio di Biotecnologie Applicate all’Ortopedia, IRCCS Istituto Ortopedico Galeazzi, 20161 Milan, Italy; 3 Unit of Clinical Epidemiology, IRCCS Istituto Ortopedico Galeazzi, 20161 Milan, Italy; 4 Department of Internal Medicine, University of Genoa, 16132 Genoa, Italy; 5 Regenerative Medicine Technologies Laboratory, Ente Ospedaliero Cantonale, Laboratories for Translational Research (LRT), 6500 Bellinzona, Switzerland; 6 Department of Surgery, Ente Ospedaliero Cantonale, Service of Orthopaedics and Traumatology, 6962 Lugano, Switzerland; 7 Faculty of Biomedical Sciences, Euler Institute, USI, 6900 Lugano, Switzerland

**Keywords:** Fetal bovine serum (FBS), Human platelet lysate (HPL), Mesenchymal stem cells (MSC), Cell proliferation, Freeze/thaw cycles

## Abstract

Mesenchymal stem cell (MSC) culturing for cell therapies needs a step forward to be routinely used in clinical settings. Main concerns regard the use of animal origin reagents, in particular supplementing the culture medium with FBS. Lately, Human Platelet Lysate (HPL) has been proposed as animal-free alternative, described as an excellent supplement for culturing MSCs. The aim of this systematic review was to analyze the current literature on the effect of HPL and FBS on ASCs and BMSCs. The primary outcome was the proliferation rate of cells cultured with FBS and HPL. Differences in terms of doubling time (DT) and population doubling (PD) were evaluated by meta-analysis, subgrouping data according to the cell type. A total of 35 articles were included. BMSCs and ASCs were used in 65.7% (23) and 28.6% (10) studies, respectively. Only two studies included both cell types. Overall, 22 studies were eligible for the meta-analysis. Among them, 9 articles described ASCs and 13 BMSCs. The results showed that BMSCs and ASCs cultured with 10% HPL and 5% HPL have lower DT and higher PD compared to cells cultured with 10% FBS. A possible correlation between the DT decrease and the application of at least 3 freeze/thaw cycles to induce platelet lysis was found. Additionally, HPL increased VEGF secretion and maintained the immuno-modulatory abilities for both cell types. The clarification reported here of the higher efficiency of HPL compared to FBS can help the transition of the scientific community towards clinical-related procedures.

**Graphical Abstract:**

1. The meta-analysis shows that HPL induces a population doubling increase and a doubling time decrease of both ASCs and BMSCs compared to FBS. 2. When at least 3 freeze/thaw cycles are applied to induce platelet lysis, the doubling time of HPL-cultured cells is lower than FBS-cultured cells (Created with BioRender.com).
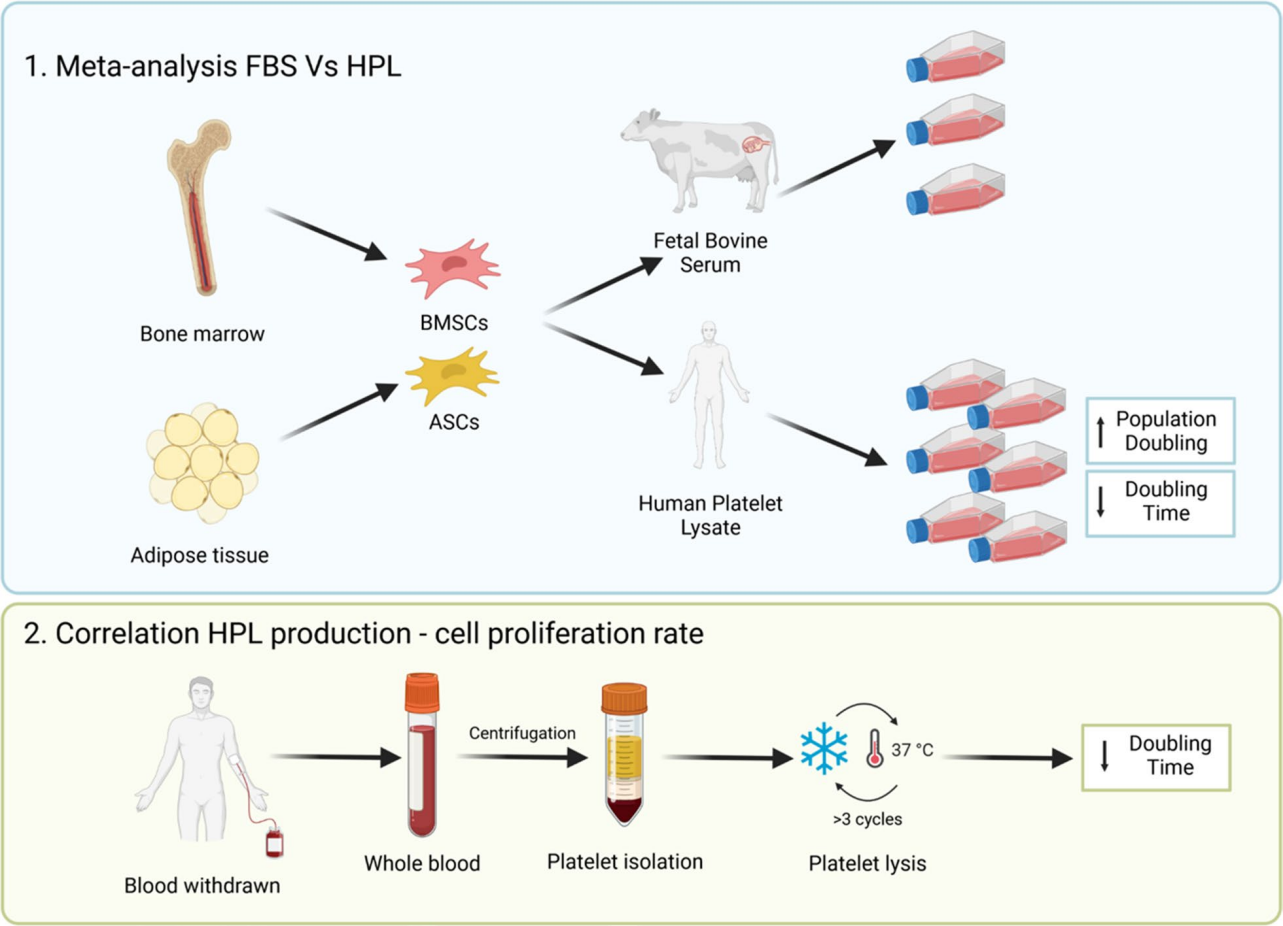

**Supplementary Information:**

The online version contains supplementary material available at 10.1186/s13287-022-02815-1.

## Background

To make mesenchymal stem cell (MSC)-based therapies a concrete clinical option, it is mandatory to implement the standardization process of the in vitro culture protocols starting from the first exploratory phases of the study. In fact, if the protocols comply with the regulatory requirements of cell-based therapy products, the evidences acquired during all the study phases can be easily translated into clinical application. In Europe, the manufacturing of MSC-based products for clinical purposes must be compliant with specific requirements set by the European Medicines Agency in the Regulation (EC) No 1394/2007 regarding Advanced Therapeutic Medicinal Products (ATMPs) (Article 17 of Regulation (EC) No 1394/2007) that demands standardized and safe protocols for cell manipulation, characterization, differentiation, and expansion during product manufacturing. Up to now, most of the MSC-related findings have been based on the use of Fetal Bovine Serum (FBS) as medium supplement for cell expansion to obtain a number of cells suitable for therapeutic use. Recently, regulatory restrictions have been raised regarding the use of FBS in clinical settings, such as the risk of xeno-immunization against bovine antigens, transmission of pathogens, and ethical problems connected with the brutal procedures used for FBS collection [1]. Therefore, the interest towards human alternatives as medium supplements has grown fast to implement the bench-to-bedside translation of MSC-based therapies.

Among them, Human Platelet Lysate (HPL) has been proposed as substitute for FBS [1] given the platelet content rich in several bioactive molecules. Large amounts of HPL can be easily obtained from unused apheresis products and buffy coats, even in the form of pooled product from different blood donors, thus reducing inter-batch variability [2]. After the first description of the preparation and use of HPL for MSC expansion by Doucet and colleagues in 2005 [3], the use of HPL has been evaluated in several in vitro and pre-clinical studies on bone-marrow- (BMSCs) and adipose-derived (ASCs) mesenchymal stem cells [4]. The common trend among these studies is that HPL seems to support cell viability similarly to FBS, without altering other features of MSCs, such as immunophenotype, clonogenic ability, and genomic stability [5, 6]. However, despite a common consensus regarding the potential of HPL, there is still large heterogeneity of production methods that inevitably leads to variable outcomes. The only common thread is the maximization of the release of growth factors by optimizing the platelet lysis [2]. Whereas, the relevance of a variety of factors possibly affecting the composition of HPL and therefore its efficacy, including donor’s features, platelet source, and lysis method [1] is generally neglected. This lack of standardized recommendations and culture protocols partly prevents the scientific community from quickly and efficiently adopting HPL for culturing MSCs.

The aim of this meta-analysis and systematic review was therefore to systematically analyze the current literature that compares the effect of HPL and FBS on ASC and BMSC cultures. Differences between HPL and FBS were firstly evaluated by a meta-analysis to understand whether HPL influenced MSC proliferation differently than FBS, in terms of doubling time (DT) and population doubling (PD). Then, differences between these two supplements were evaluated qualitatively and described through the typical MSC features (i.e. clonogenic ability, immunophenotype, morphology, mesenchymal differentiation, and immunomodulatory properties). Moreover, the presence of a possible correlation between HPL production steps and cell proliferation rate of both ASCs and BMSCs was analyzed. Based on the collected results, considerations were made on the HPL production protocols as well as on strengths and weaknesses of the use of HPL in MSC culture.

## Material and methods

### Study design

This systematic review follows the Preferred Reporting Items for Systematic Reviews and Meta-Analyses (PRISMA) checklist [7].

### Search strategy

Literature search was performed by querying three different electronic databases: PubMed, The Cochrane Library, and Embase. Study selection was executed on articles published until the end of March 2021 and without other limitations on publication period. The keyword search was structured as following: “human platelet lysate” AND “fetal bovine serum” OR “fetal calf serum” OR “FBS” OR “FCS” AND “mesenchymal stem cell” OR “mesenchymal stromal cell” OR “stem cell” OR “stromal cell” OR “progenitor cell”. The presence of all these terms was checked only within title or abstract. The full-texts of significant studies were further investigated to assess eligibility.

### Study selection

Eligibility criteria were established a priori to include journal articles in the systematic review. The retrieved articles were first screened by title and abstract. Those that satisfied the following inclusion criteria were selected: (1) description of a direct comparison between HPL and FBS for the culture of BMSCs or ASCs; (2) 2D cell-culture on plastic only (e.g. not coated); (3) English language; (4) full-text availability.

Journal articles that (1) did not include the control group (FBS-cultured MSCs); (2) used pathogen-reduced HPL; (3) declared a previous MSC contamination with animal sera (e.g. collagenase inhibition with FBS prior to culture with HPL); (4) did not provide exhaustive information on cell isolation protocol; (5) employed different basal media between FBS- and HPL-cultured cells; and (6) changed the type of medium during cell culture were excluded from the analysis. Moreover, we did not consider conference abstracts, reviews, and duplicate published data.

In the second step, the full texts of the selected articles were screened, with further exclusions according to the previously described criteria. Two investigators evaluated all the articles independently. In case of disagreement, a third investigator was consulted.

### Data extraction

Two investigators performed data extraction independently. For the included studies, relevant data were extracted from article texts, tables, and figures, and then summarized and analyzed to the purpose of the present work. In particular, the following data were collected for each study: (1) cell type (BMSCs and/or ASCs); (2) demographic features of donors; (3) isolation and expansion protocol; (4) percentage of FBS and HPL; (5) culture media and other supplements used (e.g. antibiotics and animal free trypsin); (6) proliferation rate and clonogenic/pluripotency ability; (7) immunophenotypic profile; (8) cell morphology; (9) tri-lineage mesenchymal differentiation potential; (10) safety properties (telomerase activity, karyotype analysis, etc.); (11) cell secretory activity; and (12) immunomodulatory potential. For all the analyzed records, only those experiments meeting the inclusion criteria were maintained.

The following data about HPL were also collected for each study: (1) material source used (e.g. whole blood, buffy coats and others, with the corresponding number of donors): (2) platelet lysis; (3) debris removal; (4) fibrin removal; (5) filtration; (6) storage condition; (7) any other kind of characterization and standardization.

When not clearly reported in the paper, numeric data were rigorously extracted using the online free software WebPlotDigitizer (by Ankit Rohatgi, https://automeris.io/WebPlotDigitizer) with appropriate graph settings.

### Outcomes

When culturing cells in vitro, the proliferation rate is one of the parameters that permits to evaluate the state of health of the cells. This parameter is easily calculable, objective, and allows to compare cell growing among different papers. Since the cell proliferation rate can be calculated indiscriminately as DT (the time a cell takes to double) or as PD (the number of times the cells double during the culture), we decided to consider both of them for the meta-analysis.

The primary outcome of this meta-analysis was the comparison of the proliferation rate expressed as (1) DT and (2) PD (cumulative and mean value) of cell cultured with FBS and HPL. The secondary outcome was qualitatively evaluated and performed on the following parameters: clonogenic ability (CFU-F assay), pluripotency (gene expression and immunofluorescence staining), immunophenotype (FACS), morphology (side and forward scatter, imaging), mesenchymal differentiation (staining, gene expression), cell safety (senescence, karyotype, telomerase activity), paracrine effect (ELISA assay), immunomodulatory abilities (functional assays with immune cell system).

### Risk of bias (quality assessment)

Given the lack of an official checklist for the quality assessment of in vitro studies, we defined the risk of bias for the included studies starting from the existing checklist from Cochrane [8]. We defined specific questions related to main bias categories. For each study, we considered 4 categories as sources of bias with specific questions that could be used to evaluate the quality assessment of all in vitro studies.

- Category 1: sample size and processing. Were at least 3 BMSC/ASC donors evaluated? Were all the samples treated equally regardless the experimental group?
- Category 2: suitability of the detection assay. Was the assay appropriate for the detection of the specific feature?
- Category 3: reproducibility and consistency of described methods. Were all the methods described exhaustively? Was the experimental setting performed rigorously?
- Category 4: completeness of the results. Were all the results corresponding to each method described? Were the results reported objectively?

For each bias, “yes” or “no” was attributed accordingly to its presence or absence, respectively. Moreover, “unclear” was assigned when there the elements to judge were insufficient.

### Data synthesis

Descriptive statistics were presented as median (interquartile range), mean (standard deviation) or number (percentage), depending on the analyzed parameter. Differences between FBS and HPL in terms of DT and PD from each single study were evaluated by meta-analysis subgrouping data according to the cell type, ASCs or BMSCs. Similarly, the effect of the percentage of HPL in culture medium (5% and 10%) was evaluated too. The meta-analysis was performed when outcome data were available from more than two studies, by using mean difference (MD) with 95% confidence intervals (CIs). Means were used as final values of each outcome adopting a random effects model since we expected high heterogeneity [9]. Heterogeneity was evaluated using the *I*^2^ statistic [10] where *I*^2^ > 75% is considered substantial heterogeneity. Thus, in the presence of substantial heterogeneity, we performed sensitivity analysis based on biological assumptions excluding high risk of bias related to missing or unclear methods used in the research practice (category 3-item 1) and inadequate number of cell donors (category 1-item 1). Data analyses were performed using RevMan Software 5.4, The Cochrane Collaboration, 2020. For hypothesis testing, a probability value of < 0.05 was considered as statistically significant. All statistical tests were 2-sided.

Associations between DT values and variables of HPL production protocols such as (1) the material used as platelet source (i.e. platelet apheresis, platelet rich plasma—PRP, buffy coat, whole blood), (2) the procedure used for the platelet lysis (less or more than 3 freeze/thaw cycles), and (3) the addition of heparin in the final culture medium were evaluated too. The association between the above-mentioned variables and DT was investigated with graphical tools.

Statistical analysis was performed by GraphPad Prism v7.0 (Graphpad Software). Normality of data was assessed by Shapiro–Wilk tests. Wilcoxon matched pairs signed rank test was used to compare two conditions of the same parameter (e.g. heparin addition or not). Non parametric one-way (ANOVA) by Kruskal–Wallis test and Dunn’s multiple comparisons test was used for comparison of more than two conditions (e.g. the type of starting material).

## Results

### Study selection

**Fig. 1:**
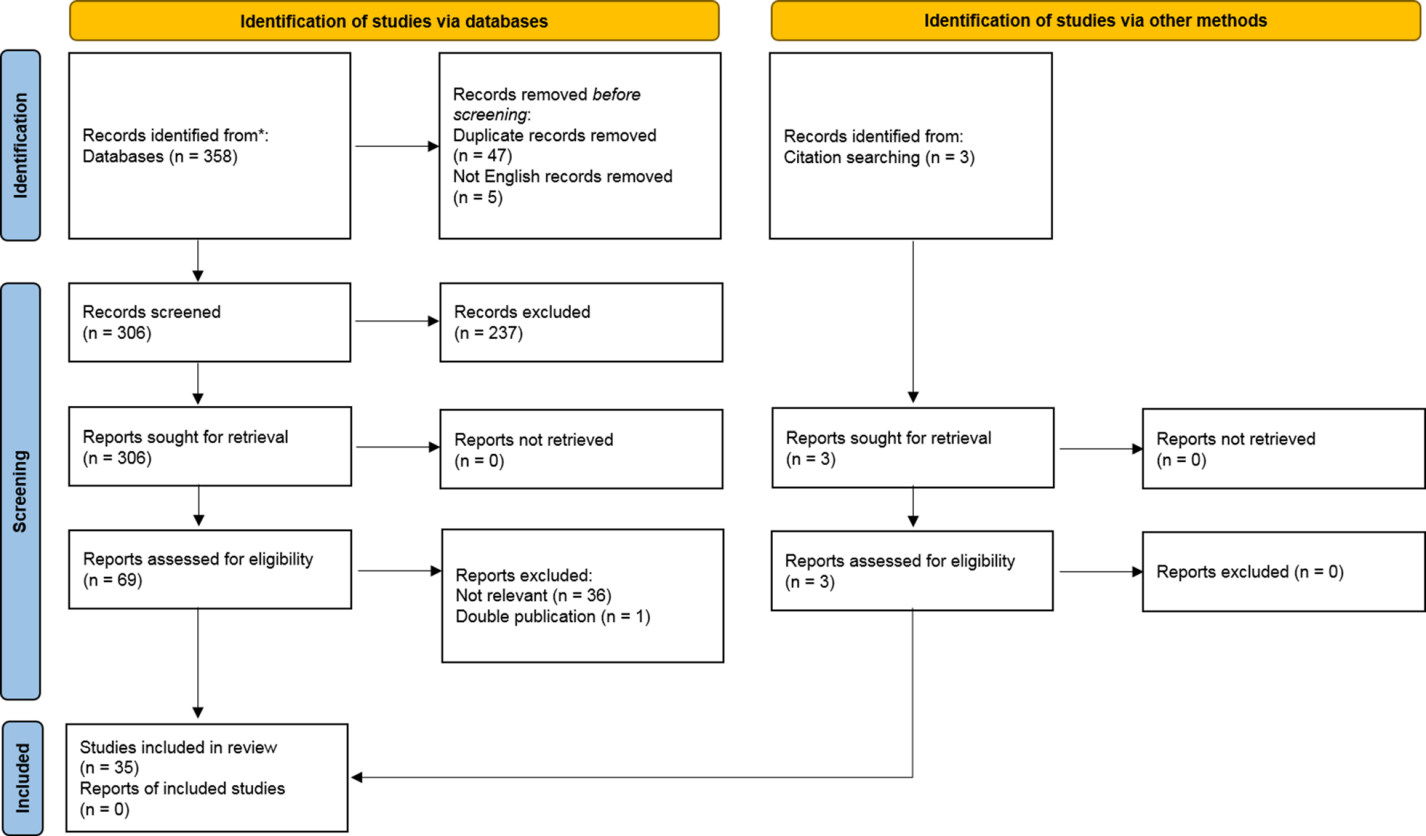
Flowchart of included articles. A total of 35 articles met the inclusion criteria after the validation process

The initial search identified 358 records. The study selection process led to the exclusion of 47 duplicate records and 5 articles written in a different language than English. Among the remaining records screened for title and abstract (*n* = 306), 6 reviews and 161 congress abstracts were excluded due to the lack of specific data and 70 because non-related records. The full-text of 69 articles was then analyzed. Among them, 36 articles that did not satisfy one or more inclusion criteria and 1 article that was a duplicate of another publication were excluded. Other 3 articles were retrieved from the bibliography of other papers, yielding a final number of 35 articles which were included in this review (Fig. 1).

### Features of included studies

The main features of the studies included are reported in Tables 1 and 2. The first paper that directly compared FBS and HPL for BMSC culture was published in 2005, while the first paper concerning ASCs was published in 2009. BMSCs and ASCs were used in about 65.7% (23/35) and 28.6% (10/35) studies, respectively, whereas only two included both cell types [11, 12]. In all the articles, 10% FBS in the culture medium was considered as the control group in this meta-analysis. Only one study described also the use of 5% and 20% FBS [13]. Conversely, the analyzed studies reported the use of various HPL concentrations. In particular, 10% and 5% HPL were applied in 23 and 16 studies, respectively. Other concentrations (0.5, 1, 2.5, 7.5, 8, 20%) were described in 7 studies articles only. Most papers used home-made HPL (31/35), whereas only 4 studies tested a commercial HPL (Table 1). Among the retrieved papers, two of them investigated the effect of autologous HPL on MSC expansion [14, 15].

**Table 1:** Main features and analysis performed in retrieved papers

Reference	Type of cells	Number of donors and age	Culture supplement		Type of HPL	Standard characterization (proliferation, immunophenotype, morphology)	Differentiation	Safety	Immunomodulatory/anti-inflammatory/angiogenic properties
			FBS	HPL					
Shanbhag et al. [11]	ASC BMSC	3 8–14 yo	10%	5%	Home-made	Proliferation (DNA quantification) Immunophenotype (FACS analysis) Morphology (qualitative)	Adipogenic (Oil Red O staining) Osteogenic (Alizarin red S Staining, qPCR, ALP activity)	–	–
Fuoco et al. [16]	ASC	2 F 53–56 yo	10%	10%	Home-made	Proliferation (DT, MTT assay) Immunophenotype (FACS analysis) Morphology (qualitative)	Adipogenic (Oil Red O staining) Osteogenic (Alizarin Red staining) Chondrogenic (Alcian Blue staining)	–	–
Palombella et al. [5]	ASC	3 m. 49 ± 2 yo	10%	5%	Heparin-free HPL, GMP Grade (Antibodies-online.com)	Proliferation (MTS assay) Immunophenotype (FACS analysis) Morphology (qualitative)	Adipogenic (Oil Red O staining) Osteogenic (Alizarin Red staining)	–	Secreted cytokines (ELISA) Neurotrophic properties (qPCR, IF, co-culture with dorsal root ganglia)
Gao et al. [17]	ASC	8 40–80 yo	10%	5% 10%	Home-made	Proliferation (DT) Immunophenotype (FACS analysis) Pluripotency (qPCR, IF) Morphology (qualitative)	Osteogenic (ALP activity, Von Kossa staining and quantification) Chondrogenic (Alcian Blue staining and quantification)	–	–
Becherucci et al. [18]	BMSC	12 m. 25 yo	10%	5%	Home-made	Proliferation (PD) Immunophenotype (FACS analysis) Morphology (qualitative, FSC, SSC)	Adipogenic (Oil Red O staining) Osteogenic (Alizarin Red staining) Chondrogenic (Alcian Blue staining)	Relative telomere length	Mixed leukocyte reaction T-regulatory cell induction
Boraldi et al. [19]	BMSC	1 M 42 yo	10%	5% 8%	Stemulate, cook medical (+ / − heparin) Macopharma Lyset (+ heparin; Sclavo Diagnostic International)	Proliferation (PD)	–	–	–
Pierce et al. [20]	BMSC (Lonza)	3	10%	10%	Home-made	Proliferation (DT, MTT assay, cell counting) Immunophenotype (FACS analysis)	–	–	Microarray gene expression analysis
Fernandez-Rebollo et al. [21]	BMSC	6 54–82 yo	10%	10%	Home-made	Proliferation (DT, PD, cell counting) Immunophenotype (FACS analysis) Morphology (qualitative, aspect ratio) Focal adhesions (IF)	Adipogenic (BODIPY/DAPI staining) Osteogenic (Alizarin Red staining)	Senescence (β-galactosidase staining) DNA-methylation analysis	Microarray gene expression analysis and semi-quantitative PCR
Frese et al. [22]	ASC	5 F m. 49 ± 8 yo	10%	10%	Home-made	Proliferation (DT) Immunophenotype (FACS analysis, IHC) Morphology (qualitative)	Adipogenic (Oil Red O staining) Osteogenic (Alizarin Red staining) Chondrogenic (Toluidine Blue staining)	–	–
Juhl et al. [23]	BMSC	1 M, 2 F 20–25 yo m. 22 yo	10%	5%	PLTMax (+ heparin) Stemulate, Cook Medical (+ / − heparin)	Proliferation (PD) Immunophenotype (FACS analysis) Morphology (qualitative)	Adipogenic (Oil Red O staining) Osteogenic (Alizarin Red staining) Chondrogenic (Alcian Blue staining)	Genomic stability (Comparative Genomic Hybridization)	–
Riis et al. [24]	ASC	5	10%	5% 10%	Stemulate, Cook Medical	Proliferation (DT, PD, CFU, cell counting) Immunophenotype (FACS analysis) Cell attachment on culture surface Morphology (qualitative, FSC, SSC)	–	–	–
Castrèn et al. [25]	BMSC	20–30 yo	10%	0.5%	Home-made	Immunophenotype (FACS analysis)	Osteogenic (ALP activity, Sirius Red staining and quantification, Alizarin Red staining and quantification, calcium content, qPCR)	–	–
Hildner et al. [26]	ASC	8	10%	5% 10%	Home-made	Proliferation (PD)	Chondrogenic (Alcian Blue staining, IHC, GAG quantification, qPCR)	–	–
Muraglia et al. [27]	BMSC	3	10%	0.5% 1% 2.5%	Home-made	Proliferation (PD, CFU, MTT assay) Immunophenotype (FACS analysis)	–	–	–
Castiglia et al. [28]	BMSC	19 0.5–39 yo	10%	10%	Home-made	Proliferation (PD, CFU) Immunophenotype (FACS analysis) Pluripotency (IF staining and quantification, qPCR) Morphology (qualitative)	Adipogenic (Oil Red O staining) Osteogenic (Von Kossa staining) Chondrogenic (Alcian Blue staining)	Karyotype analysis Tumorigenesis test	–
Fekete et al. [13]	BMSC	3	5% 10% 20%	5% 10% 20%	Home-made	Proliferation (cell counting, seeding density effect)	–	–	–
Bernardi et al. [29]	BMSC	4	10%	2.5% 5% 7.5% 10%	Home-made	Proliferation (DT, PD) Immunophenotype (FACS analysis)	–	–	–
Kinzebach et al. [12]	ASC BMSC	3 F—ASC 3—BMSC	2.5% 5% 7.5% 10%	2.5% 5% 7.5% 10%	Home-made	Proliferation (ATP content assay, protein influence) Immunophenotype (FACS analysis) Morphology (qualitative)	Adipogenic (Oil Red O staining) Osteogenic (Von Kossa staining)	–	Mixed leukocyte reaction (only ASC) Secreted cytokines (ELISA)
Menard et al. [30]	BMSC	nd	10%	8%	Home-made	Immunophenotype (FACS analysis)	–	–	Anti-inflammatory genes (qPCR) Inhibition of immune cell proliferation Mixed lymphocyte reaction IDO activity
Trojahn Kølle et al. [31]	ASC	4 F	10%	10%	Home-made	Proliferation (DT) Immunophenotype (FACS analysis) Morphology (qualitative)	Adipogenic (Oil Red O staining) Osteogenic (Alizarin Red staining) Chondrogenic (Alcian Blue staining)	Chromosomal stability analysis	In vitro angiogenesis test Secreted cytokines (ELISA)
Warnke et al. [32]	BMSC	1 M 53 yo	10%	10%	Home-made	Proliferation (WST assay) Immunophenotype (FACS analysis)	Adipogenic (Oil Red O staining) Osteogenic (Alizarin Red staining)	–	–
Azouna et al. [33]	BMSC	13 16–41 yo m. 33 ± 2 yo	10%	5% 10%	Home-made	Proliferation (DT, CFU) Immunophenotype (FACS analysis) Morphology (qualitative)	Chondrogenic (Alcian Blue staining, qPCR, WB)	–	Secreted cytokines (ELISA)
Gottipamula et al. [34]	BMSC	4	10%	10%	Home-made	Proliferation (DT, PD, CFU) Immunophenotype (FACS analysis) Morphology (qualitative)	Chondrogenic (Safranin O staining)	–	Mixed leukocyte reaction
Cholewa et al. [35]	ASC	5	10%	10%	Home-made	Proliferation (PD, CFU, MTT assay) Immunophenotype (FACS analysis) Morphology (qualitative)	Adipogenic (Oil Red O staining, qPCR) Osteogenic (Alizarin Red staining, qPCR)	Senescence (β-galactosidase staining)	–
Flemming et al. [36]	BMSC	5 21–72 yo	10%	10%	Home-made	Immunophenotype (FACS analysis) Morphology (qualitative)	–	–	Polyclonal stimulation Mixed leukocyte reaction Degranulation assay with T cells
Castegnaro et al. [37]	ASC	7 25–50 yo m. 40 yo	10%	10%	Home-made	Proliferation (PD, CFU) Immunophenotype (FACS analysis)	Adipogenic (Oil Red O staining, qPCR) Osteogenic (Von Kossa staining, qPCR)	ALDH activity	T-cells proliferation inhibition
Chevallier et al. [38]	BMSC	10 36–54 yo	10%	5%	Home-made	Proliferation (DT)	–	–	–
Horn et al. [14]	BMSC	nd	10%	10%	Home-made	Proliferation (PD, CFU, MTT assay) Immunophenotype (FACS analysis) Morphology (qualitative)	Adipogenic (Oil Red O staining, BODIPY/DAPI staining and quantification) Osteogenic (Alizarin Red staining)	Senescence (β-galactosidase staining)	–
Schallmoser et al. [39]	BMSC	1 F, 2 M 9, 27, 36 yo	10%	10%	Home-made	Proliferation (PD, CFU) Immunophenotype (FACS analysis) Morphology (qualitative)	Adipogenic (Oil Red O staining)	Senescence (qPCR)	Microarray gene expression analysis
Bieback et al. [40]	BMSC	14 m. 22 yo	10%	10%	Home-made	Proliferation (DT, PD, CFU) Immunophenotype (FACS analysis) Morphology (qualitative)	Adipogenic (Oil Red O staining) Osteogenic (Von Kossa staining)	Telomerase activity	T-cells proliferation inhibition Secreted cytokines (cytokines antibody array)
Blande et al. [15]	ASC	9 FBS, 4 HPL	10%	10%	Home-made	Proliferation (DT) Immunophenotype (FACS analysis) Morphology (qualitative)	Adipogenic (Oil Red O staining) Osteogenic (Alizarin Red staining)	–	–
Prins et al. [41]	BMSC	9 4–74 yo	10%	5%	Home-made	Proliferation (PD, CFU) Immunophenotype (FACS analysis) Morphology (FSC, SSC)	Osteogenic (ALP expression) Chondrogenic (IF)	–	–
Capelli et al. [42]	BMSC	5	10%	5%	Home-made	Proliferation (cell counting) Immunophenotype (FACS analysis) Morphology (qualitative)	Adipogenic (Sudan IV staining) Osteogenic (ALP expression)	–	Mixed leukocyte reaction PHA stimulation assay
Schallmoser et al. [43]	BMSC	2 F, 2 M 9–43 yo	10%	10%	Home-made	Proliferation (PD, CFU) Immunophenotype (FACS analysis) Morphology (qualitative)	Adipogenic (Oil Red O staining)	–	Secreted cytokines (multiplex detection)
Doucet et al. [3]	BMSC	10	10%	5%	Home-made	Proliferation (cell counting, CFU) Immunophenotype (FACS analysis)	Osteogenic (ALP activity and immunohistochemistry, Von Kossa staining)	–	Mixed leukocyte reaction

Only experiments following including criteria were recorded

ALDH: aldehyde dehydrogenase; ALP: alkaline phosphatase; ASC: Adipose-derived stem cells; DT: doubling time; FBS: fetal bovine serum; FSC: forward scatter; HPL: human platelet lysate; IF: immunofluorescence; m.: mean; PD: population doubling; SSC: side scatter

**Table 2:** Cell culture conditions of all retrieved papers

Reference	Type of cells	Enzymatic isolation, temperature and time	Base culture medium [l-Glucose] (company)	Antibiotics	FBS supplementation		HPL supplementation		Animal free trypsin
					FBS %	other declared supplements	HPL %	other declared supplements	
Shanbhag et al. [11]	ASC BMSC	0.1% collagenase type I, 37 °C for 1 h	DMEM (Invitrogen)	1% penicillin/streptomycin	10	–	5	1 IU/mL of heparin	–
Fuoco et al. [16]	ASC	0.25% collagenase I Also mechanical isolation	DMEM-F12 (Gibco)	1% antibiotic–antimycotic solution	10	–	10	–	–
Palombella et al. [5]	ASC	0.15% collagenase II, 37 °C for 1 h Also mechanical isolation	DMEM, high glucose (Gibco)	–	10	2 mM L-glutamine	5	–	TrypLe (Gibco)
Gao et al. [17]	ASC	2.4 mg/mL collagenase I, 36–38 °C for 55–65 min	DMEM, high glucose (Sigma)	–	10	–	5 10	–	–
Becherucci et al. [18]	BMSC	–	DMEM, high glucose (Invitrogen)	–	10	–	5	40 U/mL heparin	TrypLE Select (Thermofisher Scientific)
Boraldi et al. [19]	BMSC	–	alphaMEM without nucleosides (Gibco)	10 mg/mL ciprofloxacin	10	1% L-glutamine 1 U/mL heparin	8 5	1% L-glutamine 1 U/mL heparin	–
Pierce et al. [20]	BMSC (Lonza)	nd	alphaMEM (Thermo Fisher Scientific)	–	10	–	10	–	–
Fernandez-Rebollo et al. [21]	BMSC	–	DMEM, 1 g/L glucose (PAA)	1% penicillin/streptomycin	10	1% L-glutamine	10	1% L-glutamine 0.61 U heparin	–
Frese et al. [22]	ASC	0.1 mg/mL collagenase A, 37 °C for 1 h	DMEM, high glucose (Life Technologies)	1% penicillin/streptomycin 1% amphotericin B	10	1% Glutamax	10	1% Glutamax 1 U/mL heparin	–
Juhl et al. [23]	BMSC	–	alphaMEM	1% penicillin/streptomycin	10	–	5	10 U heparin	TrypLE Select
Riis et al. [24]	ASC	0.6 U/mL collagenase NB4, 37 °C for 1 h	alphaMEM (Invitrogen)	100 U/mL penicillin 0.1 mg/mL streptomycin	10	Glutamax	5	–	TrypLE (Invitrogen)
Castrèn et al. [25]	BMSC	–	DMEM low glucose	100 U/mL penicillin 100 µg/mL streptomycin	10	2 mM L-glutamine	0.5	40 UI/mL heparin	TrypLE-express (Life Technologies)
Hildner et al. [26]	ASC	1.5 mg/mL collagenase, 37 °C for 1 h	DMEM low glucose:HAM F12 1:1	1% penicillin/streptomycin	10	1 ng/mL bFGF	5 10	2 U/mL heparin	–
Muraglia et al. [27]	BMSC	–	Coon's modified Ham's F-12 (Biochrom)	100 U/mL penicillin 100 µg/mL streptomycin	10	2 mM L-glutamine 1 ng/mL FGF-2	5	2 mM L-glutamine 40 U/mL heparin	–
Castiglia et al. [28]	BMSC	–	alphaMEM (Sigma)	–	10	–	10	20 U/mL heparin	–
Fekete et al. [13]	BMSC	–	alphaMEM, 1 g/L glucose (Gibco) DMEM, 4.5 g/L glucose (Gibco) IMDM (Gibco) RPMI 1640 (Lonza)	–	5 10 20	–	5 10 20	2 U/mL heparin	–
Bernardi et al. [29]	BMSC	–	DMEM ATMP-Ready (PAA)	–	10	–	10 7.5 5 2.5	– 2.5% hSA 5% hSA 7.5% hSA	TrypLE Select (Life Technologies)
Kinzebach et al. [12]	ASC BMSC	collagenase –	DMEM (Lonza)	100 U/mL penicillin 0.1 mg/mL streptomycin	10	4 mM L-glutamine 5 U/mL heparin	10	4 mM L-glutamine 5 U/mL heparin	–
Menard et al. [30]	BMSC	–	alphaMEM	10 µg/mL ciprofloxacin (FBS) 12 µg/mL ciprofloxacin (HPL)	10	1 ng/mL FGF-2	8	2 U/mL heparin	Trypzean (Lonza)
Trojahn Kølle et al. [31]	ASC	collagenase NB4, 37 °C for 45–60 min	DMEM (PAA Laboratories)	1% penicillin/streptomycin	10	1% Glutamax	10	2 U/mL heparin	TrypLE express (Invitrogen)
Warnke et al. [32]	BMSC	–	AlphaMEM (Sigma)	1% penicillin/streptomycin	10	2 U/mL heparin 2 mM L-glutamine	10	2 U/mL heparin 2 mM L-glutamine	–
Azouna et al. [33]	BMSC	–	alphaMEM (Invitrogen)	100 U/mL penicillin 0.1 mg/mL streptomycin 25 µg/mL amphotericin B	10	2 mM L-glutamine 1 ng/mL bFGF	5 10	–	–
Gottipamula et al. [34]	BMSC	–	DMEM low glucose (Invitrogen) DMEM-KO (Invitrogen)	penicillin/streptomycin	10	2 mM glutamax	10	–	–
Cholewa et al. [35]	ASC	2 mg/mL collagenase I + 15 g/L BSA, 37 °C for 45 min	DMEM, low glucose (PAA Laboratories)	100 U/mL penicillin/streptomycin	10	2 mM L-glutamine	10	2 mM L-glutamine 2 U/mL heparin	–
Flemming et al. [36]	BMSC	–	DMEM, low glucose (Biochrom)	100 U/mL penicillin 100 µg/mL streptomycin	10	2 U/mL heparin	10	2 U/mL heparin	–
Castegnaro et al. [37]	ASC	0.1% collagenase A, 37 °C for 60 min	DMEM low glucose (Sigma)	100 U/mL penicillin 100 µg/mL streptomycin	10	4 mM L-glutamine	10	4 mM L-glutamine	–
Chevallier et al. [38]	BMSC	–	alphaMEM (Invitrogen) LP02 (MacoPharma)	0.5% ciprofloxacin	10	1% L-glutamine	5	1% L-glutamine 2 U/mL heparin	–
Horn et al. [14]	BMSC	–	DMEM low glucose (PAA Laboratories)	100 U/mL penicillin/streptomycin	10	2 mM L-glutamine	10	2 mM L-glutamine 2 U/mL heparin	–
Schallmoser et al. [39]	BMSC	–	alphaMEM (Sigma)	100 U/mL penicillin 100 µg/mL streptomycin	10	25 mM HEPES 2 mM L-glutamine	10	25 mM HEPES 2 mM L-glutamine 2 U/mL heparin	–
Bieback et al. [40]	BMSC	–	DMEM low glucose (Lonza)	50,000 U penicillin 50 µg streptomycin	10	4 mM L-glutamine	10	4 mM L-glutamine 2 U/mL heparin	–
Blande et al. [15]	ASC	0.075% collagenase IA, 37 °C for 30 min	DMEM low glucose (Gibco)	100 U/mL penicillin 100 µg/mL streptomycin	10	–	10	2 U/mL heparin	–
Prins et al. [41]	BMSC	–	alphaMEM (Life Technologies)	100 U/mL penicillin 100 µg/mL streptomycin	10	0.2 mM ascorbic acid-2-phosphate 2 mM L-glutamine 1 ng/mL bFGF	5	10 U/mL heparin	–
Capelli et al. [42]	BMSC	–	DMEM low glucose (Gibco)	0.1 mM gentamicin	10	1000 U heparin	5	1000 U heparin	–
Schallmoser et al. [43]	BMSC	–	alphaMEM (Sigma)	100 U/mL penicillin 100 µg/mL streptomycin	10	2 mM L-glutamine 25 mM HEPES	10	2 U/mL heparin	–
Doucet et al. [3]	BMSC	–	alphaMEM (ATGC Biotechnologies)	10 mg/mL ciprofloxacin	10	–	5	2 U/mL heparin	–

*nd* not defined

The enzyme collagenase was used in ASC-related studies for adipose tissue digestion (12/35). Two papers only isolated cells with a mechanical method. The culture medium for ASCs was mostly based on DMEM (9/12), followed by DMEM/F12 (2/12) and alpha-MEM (1/12). On the other hand, BMSCs were isolated with gradient centrifugation protocols (25/35) and cultured either with alpha-MEM (13/25) or DMEM (12/25). Other culture medium for BMSCs included Coon’s modified HAM F12, RPMI, IMDM or LP02 (3/25). In the 80% of studies, antibiotics were added to the culture medium (28/35): in most studies, penicillin and streptomycin were used (22/35), followed by ciprofloxacin (5/35), amphotericin B (2/35) and gentamicin (2/35). The addition of other components to culture medium, such as L-glutamine and heparin, differed basing on the use of FBS and HPL. Indeed, L-glutamine was added in FBS-based medium in 20/35 studies (57.1%), while in HPL-based medium L-glutamine was added in 12/35 of them (34.3%). Moreover, culture media containing FBS were also supplemented with (basic fibroblast growth factor) bFGF (5/35), ascorbic acid and HEPES (3/35), and heparin (5/35). Almost one third of the articles did not add any supplement other than FBS (11/35). For what concerns HPL-based medium, in order to avoid clot formation heparin was added in 26 out of 35 studies. Moreover, human Serum Albumin (1/35) and HEPES (1/35) were added to final medium. No other components besides HPL were added in 7/35 articles. Overall, only a small number of papers (8/35) declared to use animal-free origin trypsin for cell detachment (Table 2).

### Quantitative analysis of primary outcomes by meta-analysis

Most papers evaluated at least one parameter concerning cell proliferation between DT and/or PD (24/35) (Table 1). Overall, 22 studies reported data that were eligible for the meta-analysis. Among them, 9 articles reported data on ASCs and 13 on BMSCs. Data reported in one study were not considered for the meta-analysis because cells were expanded until they reached a specific PD, representing a diverging methodology from the other studies [19]. Among the studies included in the meta-analysis, only one applied autologous HPL and presented mediated results among donors [15]. The other paper using autologous HPL and presenting data divided for each donor was not considered [14].

#### Doubling time (DT)

**Fig. 2:**
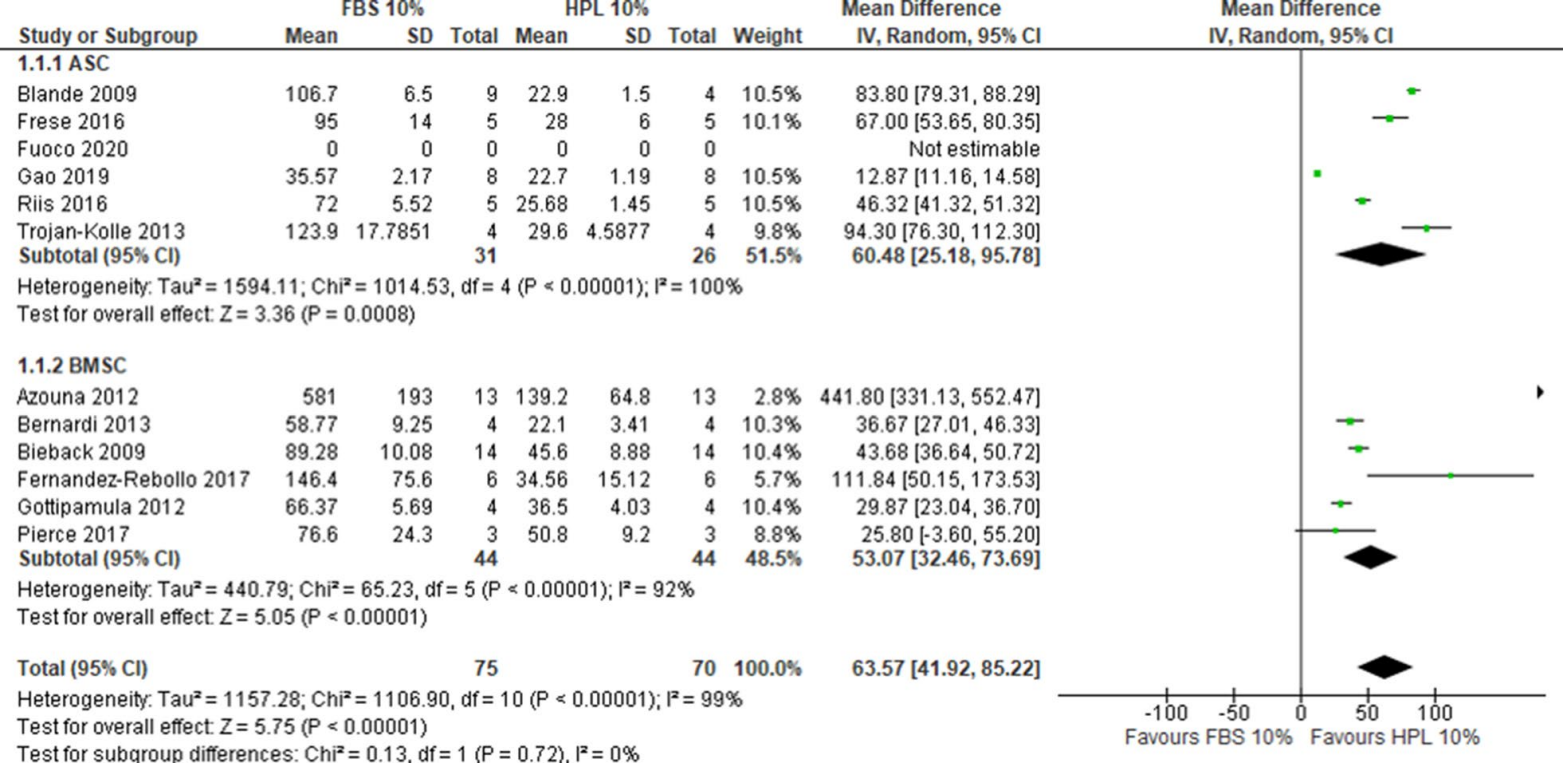
Forest plot of doubling time for FBS 10% versus HPL 10%. For both BMSCs and ASCs the DT decreased with 10% HPL compared to 10% FBS

**Fig. 3:**
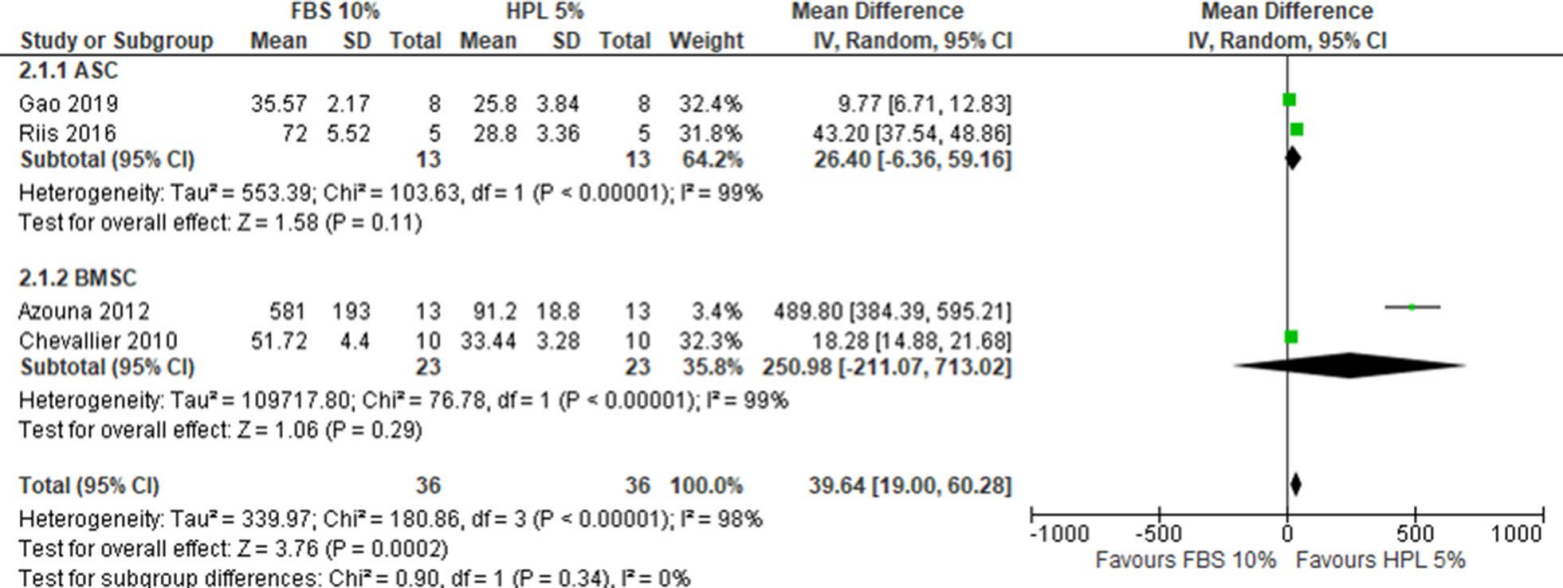
Forest plot of doubling time for FBS 10% versus HPL 5%. The supplementation with 5% HPL is slightly favored compared to 10% FBS

**Fig. 4:**
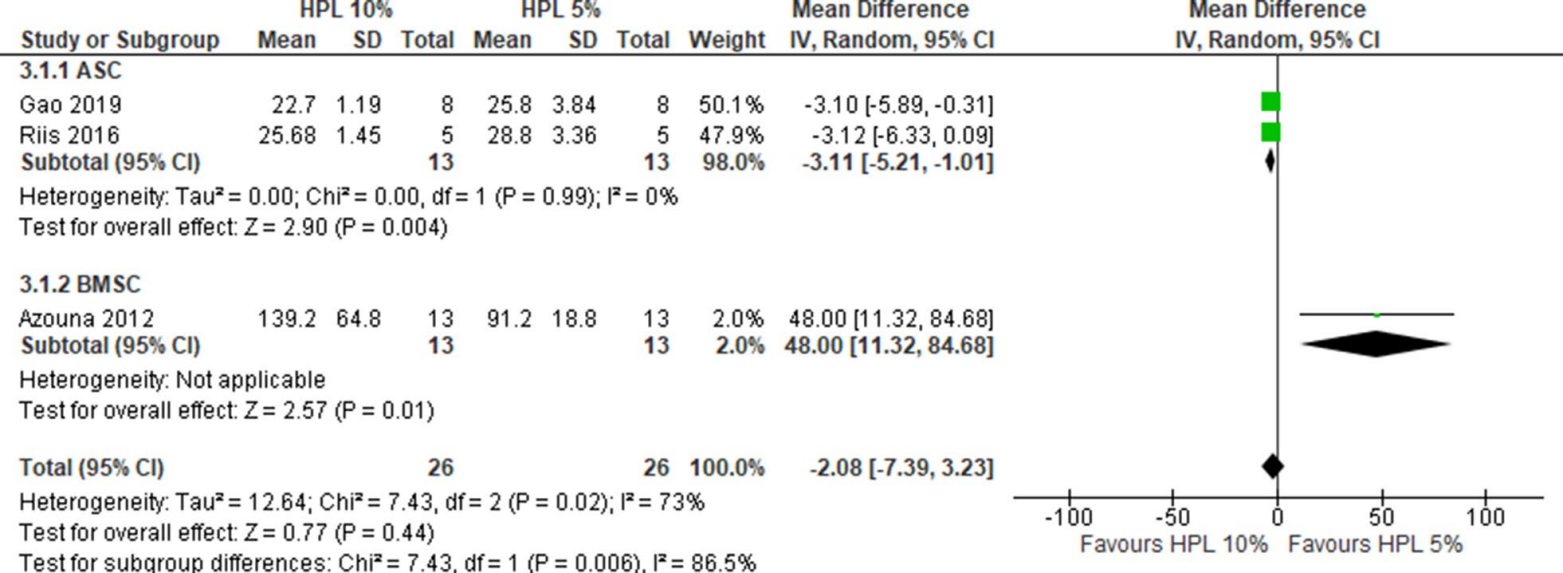
Forest plot of doubling time for HPL 10% versus HPL 5%. Only for BMSCs, the DT decreases with 5% HPL. For ASCs, there are no differences if using HPL at 5 or 10%

Overall, 12 studies reported DT data that allowed for the comparison of FBS 10% *versus* HPL 10%, of which 6 used ASCs and 6 BMSCs. One study reported the outcome but did not include data that could be used for the cumulative analysis [16]. With a very substantial heterogeneity (*I*^2^ > 90%), HPL 10% resulted in lower DT compared to FBS 10% (MD 63.57, CI 95% 41.92–85.22, *I*^2^ = 99%) (Fig. 2), corresponding to a superior proliferation rate in HPL than in FBS. A sensitivity analysis confirmed the primary analysis in favor of HPL 10% (2 studies, MD 85.28, CI 95% 78.11–92.45, *I*^2^ = 19%, Additional file 4: Fig. S1). For the comparison FBS 10% *versus* HPL 5%, data were extracted from 4 studies, equally distributed between ASCs and BMSCs. With a very substantial heterogeneity (*I*^2^ > 90%), HPL 5% resulted in lower DT compared to FBS 10% (MD 39.64, CI 95% 19.00–60.28; *I*^2^ = 98%) (Fig. 3). A sensitivity analysis confirmed the primary analysis in favor of HPL 5% (1 study, MD 9.77, CI 95% 6.71–12.83, Additional file 4: Fig. S2). For the comparison HPL 10% *versus* HPL 5%, 3 studies were included. With substantial heterogeneity (*I*^2^ = 73%), HPL 5% resulted in lower DT compared to HPL 10% (MD − 2.08, CI 95% − 7.39–3.23) (Fig. 4).

#### Population doubling (PD)

Among the studies analyzed, 9 reported data regarding PD expressed as cumulative data measured at different passages. Table 3 reports the pooled analysis with available data at each passage (e.g. P1, P2, etc.) for the following comparisons: (a) FBS 10% *versus* HPL 10%, (b) FBS 10% *versus* HPL 5%. For the comparison HPL 10% *versus* HPL 5% insufficient information was available to provide pooled analyses. The forest plots of meta-analyses are reported as Additional file 1. Overall HPL, irrespective of its percentage in the medium (i.e. 10% or 5%), showed better results than FBS 10% albeit a very substantial heterogeneity (*I*^2^ > 90%) and paucity of data (Table 3). Finally, 3 studies reported data regarding PD expressed as mean values among passages, of which 2 analyzed BMSCs [23, 43] and 1 analyzed ASCs [24]. However, no pooled analyses were performed due to the paucity of information.

**Table 3:** Values of PD

Passage (P)	Total studies	ASC results	BMSC results	Overall
*(a) Comparison: HPL 10% vs FBS 10%*				
P1	4 studies ASC: 1 study, not useful data, BMSC: 3 studies with useful data	No useful data	74 participants MD − 0.90 favor to HPL 10%, IC 95% − 2.35, − 0.55 *I*^2^ = 97%	74 participants MD − 0.90 favor to HPL 10%, IC 95% − 2.35, − 0.55 *I*^2^ = 97%
P2	5 studies ASC: 2 study, 1 with useful data; BMSC: 3 studies with useful data	30 participants MD − 4.58 favor to HPL 10%, IC 95% − 7.15, − 2.01 *I*^2^ = not applicable	74 participants MD − 2.76 favor to HPL 10%, IC 95% − 3.81, − 1.71 *I*^2^ = 88%	104 participants MD − 2.97 favor to HPL 10%, IC 95% − 3.97, − 1.97 *I*^2^ = 79%
P3	5 studies ASC: 2 study, 1 with useful data; BMSC: 3 studies, 2 with useful data	30 participants MD − 7.45 favor to HPL 10%, IC 95% − 10.17, − 4.73 *I*^2^ = not applicable	74 participants MD − 4.68 favor to HPL 10%, IC 95% − 6.05, − 3.31 *I*^2^ = 85%	104 participants MD − 5.17 favor to HPL 10%, IC 95% − 6.62, − 3.72 *I*^2^ = 81%
P4	4 studies ASC: 2 study, 1 with useful data; BMSC: 2 studies, 1 with useful data	30 participants MD − 9.66 favor to HPL 10%, IC 95% − 12.38, − 6.94 *I*^2^ = not applicable	36 participants MD − 7.16 favor to HPL 10%, IC 95% − 8.11, − 6.21 *I*^2^ = not applicable	66 participants MD − 8.07 favor to HPL 10%, IC 95% − 10.43, − 5.71 *I*^2^ = 65%
P5	3 studies ASC: 1 study with useful data; BMSC: 2 studies, 1 with useful data	14 participants MD − 14.00 favor to HPL 10%, IC 95% − 16.47, − 11.53 *I*^2^ = not applicable	36 participants MD − 10.29 favor to HPL 10%, IC 95% − 11.75, − 8.83 *I*^2^ = not applicable	50 participants MD − 12.01 favor to HPL 10%, IC 95% − 15.63, − 8.38 *I*^2^ = 84%
P7	3 studies ASC: 1 study with useful data; BMSC: 2 studies with useful data	10 participants MD − 21.00 favor to HPL 10%, IC 95% − 25.96, − 16.04 *I*^2^ = not applicable	36 participants MD − 9.63 favor to HPL 10%, IC 95% − 17.21, − 2.04 *I*^2^ = 98%	46 participants MD − 13.10 favor to HPL 10%, IC 95% − 20.48, − 5.73 *I*^2^ = 97%
*(b) Comparison: HPL 5% vs FBS 10%*				
P1	BMSC: 1 study	No useful data	24 participants MD − 0.39 favor to HPL 5%, IC 95% − 0.51, − 0.27 *I*^2^ = not applicable	No pooled analyses available
P2	2 studies ASC: 1 study with no useful data; BMSC: 1 studies with useful data	No useful data	24 participants MD − 0.89 favor to HPL 5%, IC 95% − 1.07, − 0.71 *I*^2^ = not applicable	No pooled analyses available
P3	3 studies ASC: 1 study with no useful data; BMSC: 2 studies with useful data	No useful data	42 participants MD − 1.73 favor to HPL 5%, IC 95% − 3.34, − 0.11 *I*^2^ = 83%	No pooled analyses available

### Descriptive analysis of MSC features related to medium supplementation

#### CFU-F assay

The CFU-F assay was performed in 13/35 of the studies (Table 1). Most of the papers (9/13) showed similar results between cells cultured in FBS or HPL in terms of number of colonies, whereas three studies [27, 34, 37] observed a greater number of colonies in cells cultured with HPL, and one paper [24] the opposite. More uniform data concerned the morphology of the cells forming the colonies and the size of the colonies themselves. Colony cells grown in HPL generally appeared smaller with spindle-shaped morphology and characterized by fast proliferation, indicating that the use of HPL promotes the formation of bigger colonies more densely packed with smaller cells, not observed in FBS cultures.

#### Stem cell markers expression

Only 2 papers investigated cell pluripotency by evaluating the expression of *NANOG*, *OCT4*, and *SOX2* in ASCs and BMSCs [17, 28]. Although both studies reported an increased expression of these genes in HPL-cultured cells compared to FBS, the immunofluorescence analysis revealed that the related proteins were expressed at the same level in cells cultured with FBS and HPL.

#### Immunophenotype

The immunophenotype analyzed by FACS was reported in 31/35 of the papers (Table 1). Overall, the results showed that the supplementation with HPL did not affect the expression of MSC-related markers compared to FBS culture, neither in ASCs nor in BMSCs. In fact, CD73, CD90, and CD105 were positively expressed (> 90%) whereas CD14, CD31, CD34, CD45, and HLA-DR were not or scarcely expressed (< 5%) by all groups, regardless of the culture supplement or cell type. The expression of other MSCs markers, such as CD19, CD29, CD44, and HLA-ABC, was reported only in one third of the articles and therefore it was not possible to sum up the data relative to their expression to conduct a quantitative analysis (Additional file 2).

#### Morphology

Cell morphology was described in 23/35 of the papers (Table 1). With the exception of one record [41], all the studies provided phase contrast pictures of cells cultured with both supplements. In general, HPL-cultured cells displayed a smaller and more elongated morphology, characterized by lower granularity compared to FBS-cultured cells. These features were confirmed by the decrease of forward and side scatter values analyzed with FACS in 2 papers [18, 41]. Only one study reported that HPL triggered larger and more granular cells compared to FBS-cultured cells [24]. Six papers did not report any morphologic difference due to the different culture supplements.

#### Multi-differentiation potential

The ability to differentiate toward mesenchymal lineages was assessed in 26/35 of the studies (Table 1). Both BMSC and ASC achieved an efficient adipogenic, osteogenic and chondrogenic differentiation level, regardless of the use of HPL or FBS.

Adipogenic differentiation was performed in 19/35 papers. Only 2 papers investigated the expression of adipogenic markers in ASCs [35, 37], no one in BMSCs In particular, *ADIPOQ* and *FABP4* resulted overexpressed [35] and *PPARg* downregulated in HPL-cultured cells [35, 37]. Noteworthy, 2/19 papers (1 investigating ASCs and 1 BMSCs) were in countertrend and reported a lower number of differentiated cells in HPL-cultures compared to FBS-ones [32, 37]. Remarkable, 1/19 paper investigated the effect of HPL from single donors on adipogenic differentiation and revealed that the level of differentiation was variable among the different individual HPL used [14].

Osteogenic differentiation was assessed in 21/35 papers. The evaluation of the osteogenic differentiation by ALP activity was performed in 4 papers and showed some differences based on the cell type. In fact, while ASCs in HPL-supplemented culture showed a higher ALP activity than in FBS-supplemented ones [11, 17], this was not seen for BMSCs [3, 25]. Curiously, one study reported a higher ALP activity in HPL-cultured compared to FBS-cultured BMSCs. However, no significant differences in intracellular ALP activity between BMSCs and ASCs was noticed [11].

Expression of osteogenic markers was reported in 4 papers. Curiously, *ALPL* expression was markedly decreased in ASCs but slightly increased in BMSCs when cultured in HPL [25, 37]. On the other hand, both BMSCs and ASCs differentiated in the presence of HPL overexpressed *OCN* and slightly downregulated *RUNX2* compared to the same cells differentiated in FBS [11, 25, 35, 37]. Further quantitative data, such as the quantification of stained cells or calcium deposition, resulted to be slightly superior in HPL-cultured compared to FBS-cultured cells [17, 25].

Chondrogenic differentiation was reported in 11/35 papers. Among these, only one paper reported quantitative data and evidenced that ASCs cultured in HPL contained a lower level of GAGs compared to ASCs cultured in FBS [26]. Chondrogenic marker genes such as *AGC1*, *COL9A2*, and *SOX9* were analyzed too in the same study and no differences in their expression were observed basing on the type of supplement [26].

#### Safety

Almost one third of the papers (10/35) investigated safety parameters. In particular, authors did not detect abnormal telomerase activity nor differences of the relative telomere length between HPL- and FBS-cultured cells [18, 40]. Accordingly, senescence associated genes and β-galactosidase were expressed at the same level with both culture supplements [14, 21, 35, 39]. Moreover, no signs of chromosomal alterations and tumorigenesis were retrieved after culturing cells with HPL [23, 28, 31].

#### Paracrine activity of MSCs

The data regarding the secretory profile reported in 6 papers were analyzed. Among all the investigated proteins, only two (vascular endothelial growth factor—VEGF and bFGF) were analyzed in at least two papers. Particularly, the level of VEGF was increased from 2 to 3.5 times in conditioned medium containing HPL compared to FBS [31, 40, 43]. Conversely, one paper reported a decrease of VEGF in HPL-conditioned medium [33] compared to FBS-medium. Other two papers reported a slight increase of bFGF levels in HPL-cultured cells [40, 43].

#### Immunosuppressive abilities

Approximately one-fourth of the studies (9/35) investigated whether HPL supplementation affected the immunosuppressive abilities of MSCs (Table 1). Among the variety of assays performed, the mixed lymphocyte reaction (MLR) and the T Cells proliferation inhibition were the most represented. The outcomes of the MLR assay showed that the supplementation with HPL rather than FBS did not affect the ability of MSCs to inhibit the proliferation of peripheral blood mononuclear cells (PBMCs). Similarly, the inhibitory role on T cells proliferation exerted by MSCs was not affected by HPL or FBS. One study only showed a decrease of the inhibitory effects of BMSCs on PBMCs proliferation when cultured with HPL-supplemented medium in comparison to FBS-supplemented one [34]. Moreover, another study reported a superior immunosuppressive ability against natural killer (NK) cells proliferation of BMSCs supplemented with FBS than HPL [30].

### Influence of the HPL production procedure on primary outcomes

Various protocols were reported for home-made HPL production in 31 out of 35 articles (Table 4). One third of the papers reported to use whole blood to produce HPL (10/31), whereas the source material for the other articles was PRP (7/31), platelet apheresis (7/31), or buffy coat (7/31). The number of donors ranged from 4 to 400, suggesting a great variability of protocols among laboratories. Platelet lysis was performed in different ways. Most studies (20/31) used less than three freeze/thaw cycles, while 11 between three and five freeze/thaw cycles. Sonication steps were reported in 2 records [29, 37]. A key step in the HPL production is the fibrin removal to prevent clot formation, thus avoiding the need to add porcine heparin in the culture medium. This step was achieved through the incubation with CaCl_2_ in combination or not with glass beads only in 2 papers that consistently did not include heparin in the final culture medium [17, 20]. All the other 29 records did not remove fibrin during the HPL production and 4 of them did not use heparin in the final culture medium [29, 33, 34, 37].

**Table 4:** Protocols used for the production of HPL

References	Starting material	Donor number	Preliminary steps	Dilution	Platelet lysis	Debris removal	Fibrin removal	Insoluble protein removal	Filtration step(s)	Storage	Further steps	Heparin addition	Standardization and characterization
Shanbhag et al. [11]	Whole blood-derived Platelet concentrate	Four different PCs (each PC containing buffy coats from five donors)	–	–	Multiple freeze/thaw cycles	Centrifugation 3000 g, 15 min at 4 °C	–	–	–	− 80 °C	–	1 IU/mL	1) > 2 × 10^11^ platelets 2) virological test
Fuocoet al. [16]	Platelet concentrate	4	–	–	4 freeze/thaw cycles	4 × centrifugation 3600 g, 30 min	–	–	0.22 µm filter	− 20 °C	–	2 U/mL	–
Palombella et al. [5]	Commercial: HPL FD, GMP Grade (Antibodies-online.com)	150–300	nd	–	3 freeze/thaw cycles	yes	yes	nd	nd	nd	nd	–	–
Gao et al. [17]	Platelet concentrate	Minimum 15 donors	–	–	3 freeze (− 80 °C)/thaw (37 °C) cycles	Centrifugation 3000 g, 30 min at 20 °C	Incubation with 23 mM CaCl_2_ and glass beads at 24–26 °C for 1 h Centrifugation at 6000 g, 30 min at 20 °C	Heat-inactivation at 56 °C, 30 min Centrifugation	0.2 µm filter	− 20 °C	–	–	–
Becherucci et al. [18]	Whole blood	40	Centrifugation to obtain buffy coat CompoStop Flexible kit to obtain platelet concentrate (leukocyte depletion by pressure filtration) Centrifugation 400 g, 9 min at 22 °C Centrifugation 457 g, 30 min at 4 °C Filtration	Thawed fresh-frozen AB group plasma	3 freeze/thaw cycles	2 × centrifugation 4579 g, 10 min at 20 °C	–	–	0.45 µm filter 0.2 µm filter	− 80 °C	–	40 U/mL	1.5 × 10^9^–2.4 × 10^9^ platelets/mL (before lysis) Sterility test Endotoxin test
Boraldi et al. [19]	Commercial: Stemulate, cook medical (+ / − heparin) Macopharma Lyset (Sclavo Diagnostic International)	nd	nd	nd	nd	nd	nd	nd	nd	nd	nd	1 U/mL	nd
Pierce et al. [20]	PRP (apheresis, frozen, expired ≤ 3 d)	49–109	–	–	Thawing (4 °C)	PL-P: Centrifugation 4000 g for 20 min	PL-S: Incubation with 20% (w/v) CaCl_2_ at 4 °C for 24 h Centrifugation 4000 g for 20 min	–	Details not described (proprietary process)	–	–	PL-P: 2 U/mL	Sterility test Biochemical analyses Functional assessment
Fernandez-Rebollo et al. [21]	Platelet apheresis product	At least 5	–	–	2 freeze (− 80 °C)/thaw cycles (37 °C)	Centrifugation 2600 g for 30 min	–	–	0.2 µm filter	− 80 °C	–	0.61 U	–
Frese et al. [22]	Platelet apheresis product	12	–	–	Thawing (37 °C)	Centrifugation 5000 rpm for 30 min	–	–	0.22 µm filter	− 80 °C	Centrifugation 5000 rpm for 30 min before use	1 U/mL	–
Juhl et al. [23]	Commercial: PLTMax Stemulate PL-S (heparin-required) Stemulate PL-SP (heparin-free)	PLTMax: Multiple donors Stemulate: Multiple donors	nd	nd	nd	nd	nd	nd	nd	nd	nd	10 U	PLTMax: Sterility, endotoxin, mycoplasma test BMSC growth assay Stemulate: ISO9001:2015 compliant Sterility, endotoxin, mycoplasma test BMSC growth assay
Riis et al. [20)	Commercial: Stemulate, Cook Medical	Multiple donors	nd	nd	nd	nd	nd	nd	nd	nd	nd	nd	ISO9001:2015 compliant Sterility, endotoxin, mycoplasma test BMSC growth assay
Castrèn et al. [25]	Platelet apheresis product	4	centrifugation	AB plasma	5 freeze/thaw cycles	–	–	–		–	–	40 U/mL	300 × 10^9^ platelets/each platelet unit 0.8 × 10^8^ platelets/mL final concentration BMSC growth assay
Hildner et al. [26]	Whole blood	36	Centrifugation 3939 g for 11 min to isolate buffy coat 2 h at 22 °C Centrifugation 404 g for 6 min Manual squeezing of the bag in plasma extractor to obtain PRP	–	Thawing (37 °C)	Centrifugation 2000 g for 10 min	–	–	0.22 µm filter	− 80 °C	–	2 U/mL	1–2 × 10^9^ platelets/mL (before lysis) Quantification of TGFβ1, bFGF, IGF-1, PDGF-BB
Muraglia et al. [27]	Buffy coats	300–400	Centrifugation 1100 rpm for 10 min to obtain PRP Centrifugation 2600 rpm for 20 min	Platelet Poor Plasma	3 freeze/thaw cycles	Centrifugation at high speed for 20 min at RT	–	–	–	− 20 °C	Lyophilization and restoration with sterile water before use	40 U/mL	10 × 10^9^ platelets/mL (before lysis) Endotoxin, mycoplasma test Quantification of PDGF-BB, VEGF, fibrinogen, hemoglobin
Castiglia et al. [28]	Whole blood	60	Centrifugation to isolate buffy coat Automatically separated through a leukoreduction filter (TACSI system)	AB-group plasma	3 freeze (− 35 °C)/thaw (37 °C) cycles	Centrifugation 5000 g for 8 min	–	–	–	− 35 °C	–	20 U/mL	Virological test
Fekete et al. [13]	Whole blood	12	3–22 h freezing Centrifugation to obtain buffy coat Irradiation with 30 Gy Quarantine storage − 30 °C for up to 18 months	–	Thawing at 37 °C and store at 4 °C	Centrifugation 4000 rpm for 10 min at 20 °C	–	–	0.8/0.45 μm filter 0.45/0.2 or 0.35/0.2 μm filter	− 80 °C to − 30 °C	1200 × *g* for 10 min 50,000 × *g* for 60 min	2 U/mL	At least 10^9^ platelets/mL Sterility test Endotoxin test
Bernardi et al. [29]	PRP	4–6	–	–	Sonication by ultrasound stimulation at 20 kHz for 30 min OR 3 freeze (− 80 °C)/thaw cycles (37 °C)	Centrifugation 1600 g for 15 min at RT	–	–	70 µm cell Strainer	− 20 °C	–	–	Quantification of PDGF-AB
Kinzebach et al. [12]	Buffy coat	8	–	AB plasma	1 freeze (− 30 °C)/thaw (37 °C) cycle	Centrifugation 2000 g for 20 min at RT	–	–	0.45 µm filter (complete medium)	− 30 °C	Centrifugation 2000 g for 10 min	5 U/mL	–
Menard et al. [30]	Buffy coat	4	storage at 22 °C for no longer than 5 days	–	Freeze (− 30 °C)/thaw 37 °C	Centrifugation 4000 rpm 10 min at 20 °C						2 U/mL	Sterility test Endotoxin test
Trojahn Kølle et al. [31]	Buffy coat	40	Dilution and freeze (− 40 °C)	AB plasma	Thawing (37 °C)	Centrifugation 4000 g for 15 min	–	–	–	− 80 °C	–	2 U/mL	–
Warnke et al. [32]	Platelet apheresis product	10	Freeze (− 80 °C)	–	Thawing (37 °C for 10 min)	Centrifugation 4000 g for 15 min at RT	–	–	–	− 80 °C	–	2 U/mL	–
Azouna et al. [33]	Whole blood	at least 10	Incubation at 22 °C for 16 h Centrifugation 4250 g for 13 min at 22 °C to obtain buffy coat Centrifugation 341 g for 6 min at 22 °C White blood cell depletion Freeze (− 30 °C)	AB plasma	Thawing (37 °C)	Centrifugation 1400 g for 20 min	–	–	0.22 µm filter	− 80 °C	Centrifugation 4000 g for 15 min	–	Platelet concentration White/red blood cell contamination Sterility test
Gottipamula et al. [34]	Platelet concentrate	30	–	–	5 freeze/thaw cycles	Centrifugation 4000 g for 15 min	–	–	–	–	–	–	–
Cholewa et al. [35]	Platelet concentrate	5	Supplementation with acid-citrate-dextrose (1:1, v/v)	–	2 freeze (− 80 °C)/thaw (37 °C) cycles	Centrifugation 2600 g for 30 min at 4 °C	–	–	0.22 µm filter	− 80 °C	–	2 U/mL	1–2.1 × 10^9^ platelets/mL (average in platelet units)
Flemming et al. [36]	Whole blood	40	Isolation of buffy coat Centrifugation 340 g for 6 min at 22 °C Filtration for leukocyte depletion	AB plasma	1 freeze (− 30 °C)/thaw (37 °C)	–	–	–	–	− 30 °C	Centrifugation 4000 g for 15 min at RT	2 U/mL	–
Castegnaro et al. [37]	Pooled AB plasma	5	Centrifugation 240 g for 10 min	–	Freeze and sonication	Centrifugation 1500 g for 30 min	–	–	–	− 20 °C	–	–	0.686 × 10^9^ platelets/mL (average in pooled plasma)
Chevallier et al. [38]	Platelet apheresis product	4	–	–	Freeze − 80 °C	Centrifugation 1400 g	–	–	–	–	–	2 U/mL	> 1 × 10^9^ platelets/mL (in initial apheresis product)
Horn et al. [14]	Platelet concentrate	autologous	Supplementation with acid-citrate-dextrose (1:1, v/v)	–	2 freeze (− 80 °C)/thaw (37 °C) cycles	Centrifugation 2600 g for 30 min	–	–	0.2 µm filter	− 80 °C	2 U/mL	2 U/mL	1–2.1 × 10^9^ platelets/mL (average in platelet concentrates)
Schallmoser et al. [39]	Whole blood	at least 40	Incubation at 22 °C for 16 h Centrifugation 4250 g for 13 min at 22 °C to obtain buffy coat Centrifugation 341 g for 6 min at 22 °C White blood cell depletion Freeze (− 30 °C)	AB plasma	Thawing (37 °C)	Centrifugation 1400 g for 20 min	–	–	–	− 80 °C	–	2 U/mL	–
Bieback et al. [40]	Buffy coat	40–50	Centrifugation 340 g for 6 min at 22 °C Inline filtration for leukocyte depletion Freeze (− 30 °C)	AB plasma	Thawing (37 °C)	Centrifugation 1400 g for 20 min	–	–	–	− 30 °C	Centrifugation 4000 g for 15 min	2 U/mL	–
Blande et al. [15]	Whole blood	autologous	–	–	4 freeze (− 80 °C)/thaw (37 °C) cycles	4 × Centrifugation 3313 g for 30 min	–	–	0.22 µm filter	− 20 °C	–	2 U/mL	0.91 × 109 platelet/mL (average in whole blood)
Prins et al. [41]	Platelet apheresis product	5	–	–	Freeze − 80 °C	–	–	–	–	–	Centrifugation 750 g for 10 min	10 U/mL	1 × 10^9^ platelet/mL (average in platelet products)
Capelli et al. [42]	Whole blood	1	Light-spin centrifugation to isolate PRP Heavy-spin centrifugation to concentrate platelets	–	Freeze − 40 °C	3000 rpm for 10 min	–	–	–	− 20 °C	–	1000 U	1.2 ± 0.4 × 10^9^ platelets/mL (average in platelet concentrate)
Schallmoser et al. [43]	Whole blood	40	Incubation at 22 °C for 16 h Centrifugation 4250 g for 13 min at 22 °C to obtain buffy coat Centrifugation 341 g for 6 min at 22 °C White blood cell depletion Freeze (− 30 °C)	AB plasma	Thawing (37 °C)	Centrifugation 1400 g for 20 min	–	–	0.22 µm filter	− 30 °C	–	2 U/mL	Platelet concentration White and red blood cell contamination Sterility test
Doucet et al. [3]	Platelet apheresis product	10	–	–	Freeze − 80 °C	Centrifugation 900 g	–	–	–	–	–	2 U/mL	1 × 10^9^ platelets/mL (in initial apheresis product)

*nd* not defined

**Fig. 5:**
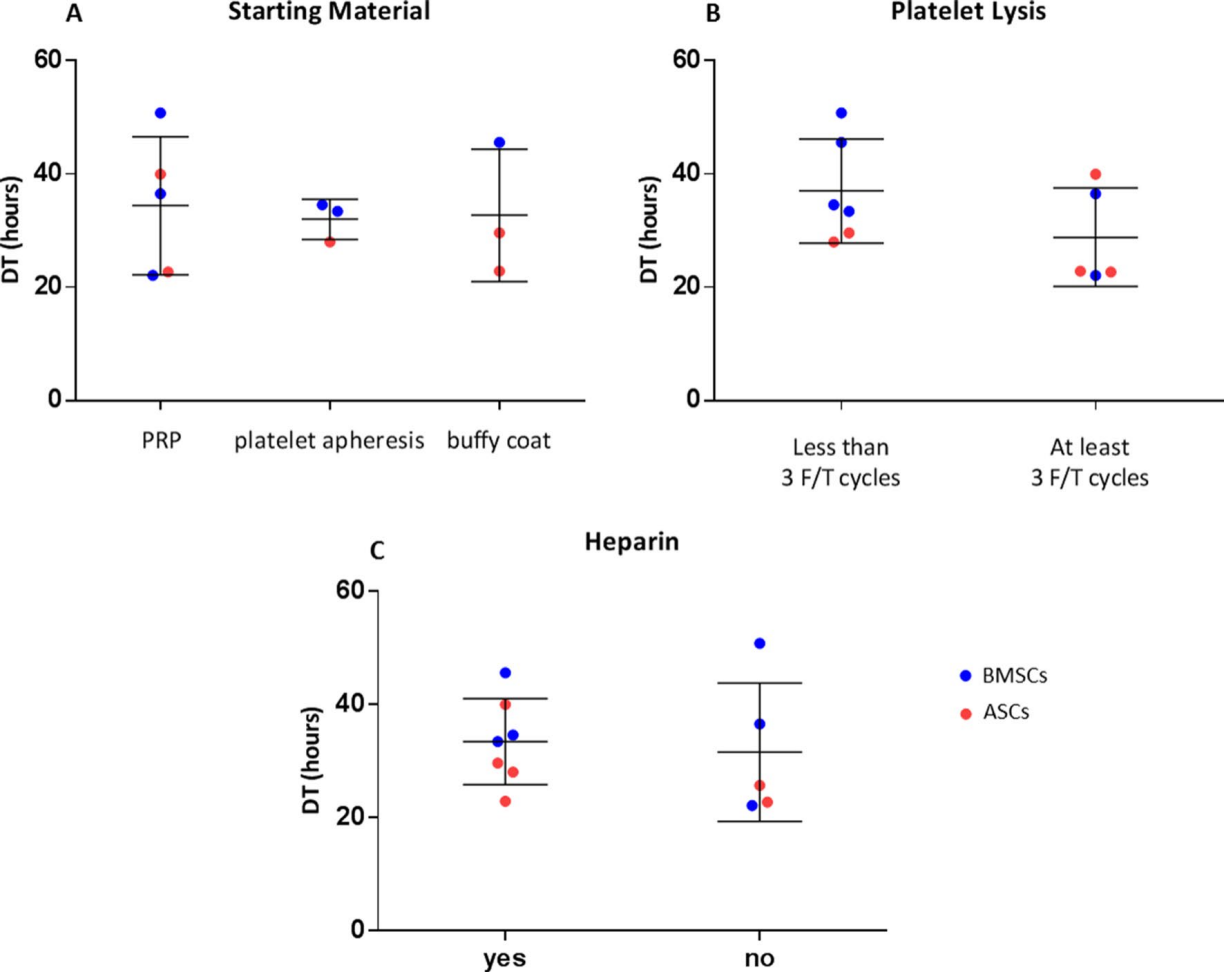
Evaluation of parameters influencing HPL effect on DT. Estimation of the effect of the starting material used for the initial isolation of platelets (PRP, platelet apheresis, buffy coat) (**A**), the method for the platelet lysis (less than 3 freeze/thaw cycles, at least 3 freeze/thaw cycles) (**B**), and the addition of heparin in the final culture medium (**C**)

Considering the large heterogeneity in term of protocols for HPL production, we investigated a possible correlation between the variables of production and the primary outcome defined as DT. Among the variables considered, the source material, the procedure for platelet lysis, and the addition of heparin in the final culture medium were the ones reporting enough data to be compared. In particular, the source material and the heparin addition had no effect on the average DT (Fig. 5A, C). It was not possible to evaluate the influence of whole blood as platelet source since the DT was reported only in one paper. Conversely, the DT seemed to be influenced by the number of cycles used for platelet lysis. In facts, papers applying less than 3 freeze/thaw cycles reported a higher DT, corresponding to slower cell proliferation, compared to papers employing 3 or more freeze/thaw cycles, albeit this trend was not statistically significant (Fig. 5B). Moreover, we did not note any clustering related to the type of cells. Notably, one record was not considered since the DT values reported were outliers [33].

### Risk of bias across studies

Overall, the 35 articles included showed moderate quality. A complete list indicating the specific bias for each study is reported in Additional file 3. The main bias retrieved were:

- Category 1—sample size and processing: Sample size of almost each study included more than 3 donors. Four studies included less than 3 donors and three studies did not specify the sample size. Sample processing was performed in the same way regardless of the experimental group, with only some bias concerning the time points considered related to the different rate in reaching cell confluence among FBS and HPL.
- Category 2—suitability of the detection assay: As for the detection methods, 6 papers investigated cell proliferation by measuring cell metabolic activities by assays such as MTT or MTS, which was considered as a bias since cell metabolic activity cannot be considered as a direct measure of cell number.
- Category 3—reproducibility and consistency of described methods: A consistent lack in the description of protocols was found. Indeed, 25 papers out of 35 did not report detailed information in their material and method section to make the procedures reproducible by third parties. As for the experimental setting, the most common bias was related to the normalization of gene expression analysis. In fact, stability tests on reference genes were generally lacking which somehow penalizes the consistency of the data reported.
- Category 4—completeness of the results. Another common bias retrieved in 16 papers was the lack of some results that were anticipated in the method section. Lastly, data from 22 papers were partially reported as raw number, thus preventing the comparison among studies.

## Discussion

Considering the potential risks of zoonoses transmission and anaphylactoid reactions related to FBS after injection in human patients [4], xenogeneic-free alternatives for MSC expansion intended for medicinal products are strongly encouraged by European Medicine Agency (EMA) (EMEA/CHMP/410869/2006). Among all the alternatives proposed, the use of HPL is progressively increasing among the cell factories. However, to date its use for research purposes is still rare as hampered, among other reasons, by the lack of definitive evidences regarding its superiority over FBS and the lack of standardized protocols defining its use. For these reasons, we systematically reviewed the literature related to in vitro studies involving MSCs from adipose tissue and bone marrow, highlighting similarities and differences deriving from the use of HPL and FBS.

The results of this systematic review first highlight that both BMSCs and ASCs proliferate faster when cultured in HPL-supplemented media than in FBS, regardless of its concentration. Specifically, both BMSCs and ASCs have a lower DT and a higher PD when cultured in HPL than FBS, allowing to obtain a higher amount of MSCs compared to FBS thus reducing the culture period. Importantly, it is clearly demonstrated that both cell types maintain unaltered features, such as the immunophenotype, the differentiation potential, the safety, and the immunosuppressive abilities regardless the type of supplement used. Conversely, other features such as the clonogenic potential, the pluripotency gene expression, and the secretion of active factors are slightly modified by the presence of the HPL. Moreover, both cell types are smaller and more spindle-shaped when cultured in HPL. Finally, the data analyzed show that the cell growth is positively influenced when platelet lysis is performed with at least three freeze/thaw cycles, providing an indication about the importance of the procedure chosen for the platelet lysis step to increase the cell performance.

Usually, the frequency of MSCs ranges from 1 to 10% of the total nucleated cells isolated [44]. Here, we summarize that the supplementation with HPL has no influence on this frequency in both ASCs and BMSCs compared to FBS as evidenced by the same number of colonies. However, colonies are heterogeneous and display different sizes and cell distribution. Indeed, given the higher DT and the lower PD in HPL-cultured cells, the studies analyzed showed that the colonies obtained in the presence of HPL are more densely populated and bigger than FBS-cultured cells. Moreover, HPL induces the increase of the expression of genes involved in the regulation of pluripotency and self-renewal pathways, such as *NANOG*, *OCT4*, and *SOX2*, suggesting that it enhances the stem potential [45].

One of the main issues related to MSC-based therapies is the possibility of tumor formation in a long-term evaluation [46]. Albeit the increase of the proliferation rate and the stem potential, no differences were observed between HPL- and FBS-cultured cells in terms of cell senescence, karyotype, and telomerase activity among the records analyzed here. However, this particular aspect is still poorly investigated when using HPL supplement, therefore additional evaluation would be needed to confirm the safety of MSCs supplemented with HPL and expanded ex vivo*.*

Our results evidenced that ASCs and BMSCs cultured with HPL secrete a higher level of VEGF and bFGF compared to counterparts cultured with FBS. This particular data is interesting as an increasing number of evidences highlights that MSCs exert their therapeutic potential mainly through the paracrine effect of extracellular vesicles and biologically-active secreted factors that stimulate survival and proliferation of resident cells, promote angiogenesis and modulate the cell immune response [47]. In particular, the initial growth of the new vascular network driven by pro-angiogenic factors represents a crucial point to obtain a successful tissue regeneration [48]. Therefore, an increase of the secretion of VEGF in MSCs supplemented with HPL represents a great advantage compared to classic procedure of FBS-based culture.

Another fundamental feature of MSCs is their ability to evade host immune system. The culture of ASCs or BMSCs with HPL maintained the ability to suppress the proliferation of T cells at comparable levels of FBS-cultured cells. The ability to bypass host immune system is mainly due the low expression level of MHC-ABC on their surface, making them invisible to NK cells. Moreover, MSCs do not express MHC-DR proteins, thus impeding the identification by CD4^+^ T cells [46]. Here, we observed that the expression of histocompatibility complex molecules is not modified when MSCs are cultured in HPL compared to FBS, therefore the shifting toward this human supplement does not hamper MSC therapy success. Along with this, MSCs possess a broad immunoregulatory ability and can suppress immune cells involved in innate and adaptive immune systems [49]. This unique immunologic abilities were firstly discovered in 2002 by demonstrating that the proliferation of leukocytes was suppressed when co-cultured with BMSCs in a dose-dependent manner [50]. Thus, MSCs exert their effect through both paracrine and cell-to-cell action [49], secreting different soluble factors some of which inhibit T cell proliferation and activation, such as hepatocyte growth factor (HGF), transforming growth factor (TGF)-β1, IL-10, and prostaglandin E2 [46]. Moreover, the lack of expression of co-stimulatory molecules, such as CD80 and CD86, is maintained in HPL-cultured cells as well as with FBS and does not provide a secondary signal during MSC-T cell interaction, thus suppressing T cell activation [49].

Albeit HPL reduces the immunologic risks linked to FBS, its human origin arises other issues, such as the transmission of blood-borne viruses, when it is obtained as a pooled product from multiple donors [28]. Autologous HPL can represent an alternative to avoid these side effects. However, autologous HPL could bring also some drawbacks. Indeed, each batch of autologous HPL can have different characteristics due to the features of individual donors, such as age, gender, and thrombocythemia. As evidenced in two retrieved papers, the differences among individual batches of HPL can result in significantly different proliferation rate, cell morphology, and adipogenic differentiation level [14, 15]. Hence, albeit cell response to autologous HPL is still superior compared to the culture with FBS, the donor-related variability of individual HPL batches may have an impact on the outcome of cell therapies, reducing the reproducibility of the procedure.

One of the main hurdles to the shift from FBS to HPL is represented by the lack of recommendations on the protocol of production of HPL together with the concentration of use in MSC cultures. Indeed, its composition and bioactivity could possibly be influenced by several factors of the production process, such as source material, platelet count, lysis process, and other [40]. Up to now, a common consensus on HPL preparation and analysis is still missing, therefore the American Association of Blood Banks and the International Society of Cell Therapy are attempting to overcome this issue by establishing quality control criteria and by standardizing the manufacturing process [40].

Different donor features, such as gender and age, could influence the final HPL product and, subsequently, the MSC response. For example, female donors could present antibodies raised against human leukocyte antigens and human neutrophil antigens from previous pregnancies, thus creating unexpected cell responses [2]. Moreover, since aging is a systemic process, platelets could also be influenced by donor age. Indeed, it was demonstrated that MSC proliferation was significantly higher when cells were cultured with HPL obtained from donors < 35 years compared to HPL obtained from donors > 45 years [51]. This same study highlighted also that HPL from older donors increased the activity of senescence-associated β-galactosidase and decreased osteogenic differentiation ability. Conversely, CFU frequency, immunophenotype, and adipogenic differentiation were not influenced by the age of HPL donors. Curiously, donor age did not influence the concentration of the main growth factors composing HPL, including PDGF, TGFβ1, bFGF, IGF-1 [51]. Considering the impact of each donor feature on the final product, the use of pooled HPL appears as a relevant strategy to reduce the age-associated differences.

Different raw materials are used as platelet sources, including buffy coat, platelet-rich plasma and apheresis products [39]. In our analysis, we did not reveal any difference on proliferation rate when MSCs were cultured with HPL obtained from different sources, even though more in-depth studies would be needed, also considering the interval between blood collection and processing. Among the different protocols for platelet lysis, freeze/thaw cycles is the most common one given its simplicity and cost-effectiveness [2]. However, the number of cycles reported in the analyzed papers ranges from 1 to 5 in absence of shared guidelines. A low number of freeze/thaw cycles may not be enough to lyse all the platelets and release all the growth factors, whereas an excessive number of freeze/thaw cycles could degrade the platelet factors [40]. Data from this review highlight a correlation between the number of freeze/thaw cycles and the cell proliferation rate measured as DT. In particular, the analysis performed showed that DT decreased with the application of at least 3 freeze/thaw cycles to induce platelet lysis. Albeit not statistically significant, this observation confirms previous evidences attesting that there was no difference on MSC proliferation when comparing one and two freeze/thaw cycles [13]. On the other hand, the comparison between two and five freeze/thaw cycles attested that the DT of cells was shorter when two cycles were used [52]. In order to better define the optimal number of cycles leading to the maximum yield of growth factor releasing more direct comparisons of different freeze/thaw cycle number would be necessary.

The presence of fibrinogen in platelets is considered a limiting factor as it can lead to the formation of clots in the final formulation of HPL and therefore can cause changes of the concentration of factors and other solutes. Adding heparin to the culture medium helps to solve this problem, however it is isolated from porcine tissues and can therefore induce hypersensitivity reactions in patients being treated [40]. Moreover, high heparin concentrations interfere with cell proliferation and possibly impede the homing of BMSCs to wounds [40]. Conversely, our literature analysis showed no influence on the proliferation rate determined by the addition of heparin.

Albeit conducted in the most rigorous way, this meta-analysis has some limitations. First, data collection was arduous given the lack of numerical results within some of the papers, thus requiring data extrapolation from graphs using an online software tool (https://automeris.io/WebPlotDigitizer). Secondly, we are aware that in the literature, it is present a great heterogeneity in terms of presenting the data and performing in vitro protocols. This great variability affects also HPL production methodology that encompasses a wide range of parameters. These limitations are due to a general lack of standardization and guidelines that characterizes many of the in vitro procedures used in this context. All these shortcomings perfectly reflect the difficulties in switching from FBS to HPL.

## Conclusions

Despite all the variables, our meta-analysis clearly demonstrates that the use of HPL increases cell proliferation rate and decreases the DT of MSCs compared to FBS culture. A faster proliferation rate is crucial to reduce expansion time and costs, thus allowing more patients to have access to biological therapies. At the same time, HPL seems not to impair other important features of MSCs. However, further studies may be required to highlight possible hidden effects. Considering the current literature, other issues such as the optimal protocol for HPL production, the need of heparin or serum-converted HPL and the relevance of the starting material on the final HPL effect on MSC culture remain partially unclear. Even though there are still many things to be defined, the observations reported by this review further encourage the shift from the use of FBS to HPL and represent a first step towards identifying recommendations for the use of HPL since the initial phases of the in vitro research.

## Supplementary Information

**Additional file 1.** Forest plots of cumulative population doubling for FBS versus HPL at different cell passage for both BMSCs and ASCs.

**Additional file 2.** Expression of surface markers evaluated by FACS.

**Additional file 3.** Risk of bias across the studies.

**Additional file 4.** Sensitivity Analysis—Forest plots of doubling time for FBS 10% versus HPL 10% and FBS10% versus HPL 5%.

## Data Availability

The datasets generated and analyzed during the current study are available in the OSF repository (https://osf.io/s98rw/?view_only=03b6831291f5407192e453f0418431eb).

## Abbreviations

ALP: Alkaline phosphatase
ASCs: Adipose-derived mesenchymal stem cells
BMSCs: Bone-marrow-derived mesenchymal stem cells
bFGF: Basic fibroblast growth factor
CFU-F: Colony-forming unit fibroblast
CI: Confidence intervals
DT: Doubling time
ELISA: Enzyme linked immunosorbent assay
EMA: European Medicine Agency
FACS: Fluorescence-activated cell sorting
FBS: Fetal Bovine Serum
HGF: Hepatocyte growth factor
HPL: Human Platelet Lysate
MD: Mean difference
MLR: Mixed lymphocyte reaction
MSCs: Mesenchymal stem cell
NK: Natural killer
PD: Population doubling
PBMCs: Peripheral blood mononuclear cells
PRP: Platelet rich plasma
TGF: Transforming growth factor
VEGF: Vascular endothelial growth factor
