# Supplementary material for: Systematic review and meta-analysis on the use of human platelet lysate for mesenchymal stem cell cultures: comparison with fetal bovine serum and considerations on the production protocol
Source: Stem Cell Res Ther. 2022 Apr 4;13:142. doi: 10.1186/s13287-022-02815-1 (PMC8981660; doi:10.1186/s13287-022-02815-1)
Supplement: Supplementary file 1 — Additional file 1. Forest plots of cumulative population doubling for FBS versus HPL at different cell passage for both BMSCs and ASCs. [file 13287_2022_2815_MOESM1_ESM.docx]

# FBS vs HPL – P1

**COMPARISON 1. FBS 10% vs HPL 10%**


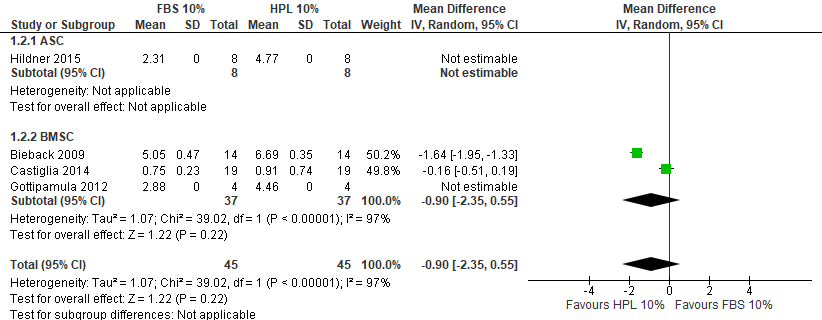


**COMPARISON 2. FBS 10% vs HPL 5%**

No sufficient studies with available data for meta-analysis.

**COMPARISON 3. HPL 10% vs HPL 5%**

No sufficient studies with available data for meta-analysis.

# FPL VS HPL - P2

**COMPARISON 1. FBS 10% vs HPL 10%**


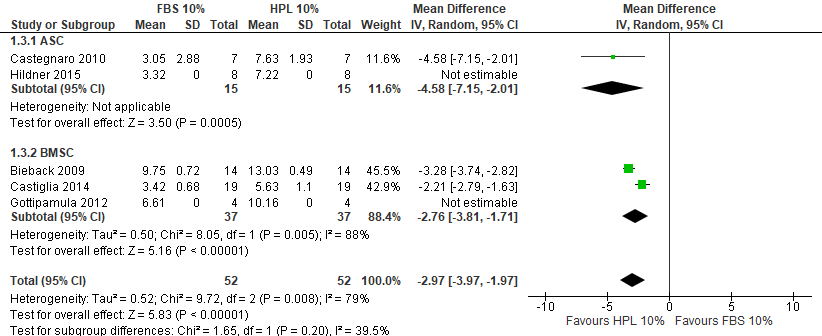


**COMPARISON 2. FBS 10% vs HPL 5%**

No sufficient studies with available data for meta-analysis.

**COMPARISON 3. HPL 10% vs HPL 5%**

No sufficient studies with available data for meta-analysis.

# FPL VS HPL – P3

**COMPARISON 1. FBS 10% vs HPL 10%**


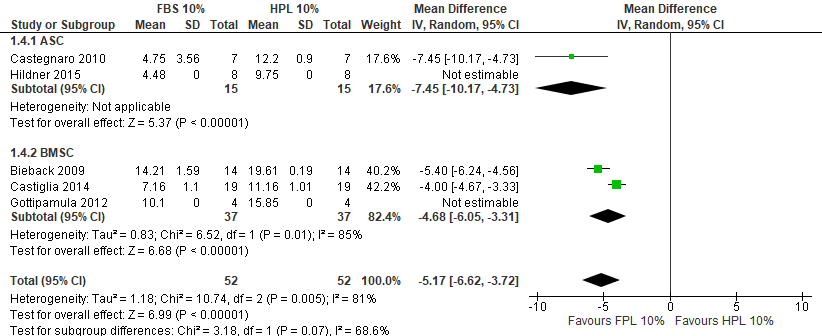


**COMPARISON 2. FBS 10% vs HPL 5%**

**
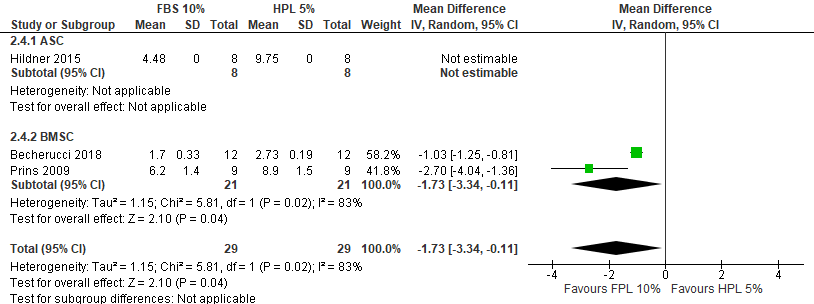
**

**COMPARISON 3. HPL 10% vs HPL 5%**

No sufficient studies with available data for meta-analysis.

# FPL VS HPL – P4

**COMPARISON 1. FBS 10% vs HPL 10%**

**
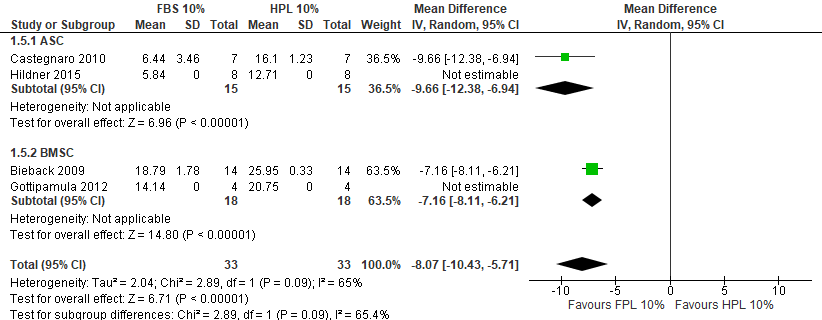
**

**COMPARISON 2. FBS 10% vs HPL 5%**

No sufficient studies with available data for meta-analysis.

**COMPARISON 3. HPL 10% vs HPL 5%**

No sufficient studies with available data for meta-analysis.

# FPL VS HPL – P5

**COMPARISON 1. FBS 10% vs HPL 10%**

**
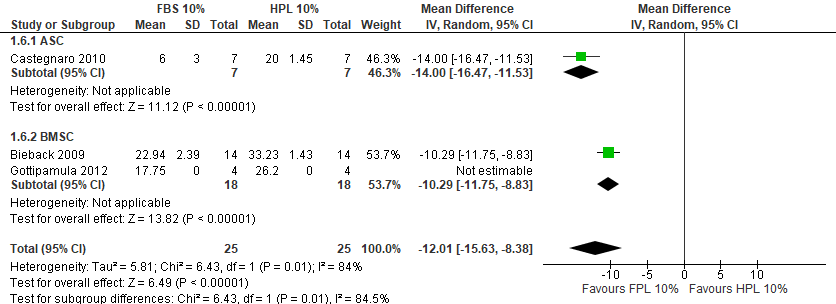
**

**COMPARISON 2. FBS 10% vs HPL 5%**

No studies included

**COMPARISON 3. HPL 10% vs HPL 5%**

No studies included

# FPL VS HPL – P7

**COMPARISON 1. FBS 10% vs HPL 10%**

**
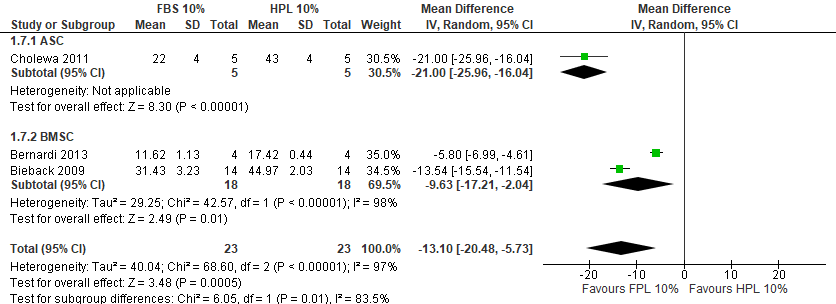
**

**COMPARISON 2. FBS 10% vs HPL 5%**

No studies included

**COMPARISON 3. HPL 10% vs HPL 5%**

No studies included
