# Supplementary material for: Systematic review and meta-analysis on the use of human platelet lysate for mesenchymal stem cell cultures: comparison with fetal bovine serum and considerations on the production protocol
Source: Stem Cell Res Ther. 2022 Apr 4;13:142. doi: 10.1186/s13287-022-02815-1 (PMC8981660; doi:10.1186/s13287-022-02815-1)
Supplement: Supplementary file 2 — Additional file 2. Expression of surface markers evaluated by FACS. [file 13287_2022_2815_MOESM2_ESM.docx]

**Additional Table 1.** Expression of surface markers evaluated by FACS

| **Negative expression** | **Positive expression** | **Unclear expression** |
| --- | --- | --- |
| CD3 | CD13 | CD4 |
| CD5 | CD49 | CD20 |
| CD10 | CD55 | CD49e |
| CD11b | CD166 | CD86 |
| CD15 |  | CD106 |
| CD33 |  | CD117 |
| CD54 |  | CD146 |
| CD56 |  | CD200 |
| CD71 |  | CD271 |
| CD80 |  | CD274 |
| CD133 |  |  |
| CD144 |  |  |
| CD235a |  |  |
| CD309 |  |  |
