# Supplementary material for: Systematic review and meta-analysis on the use of human platelet lysate for mesenchymal stem cell cultures: comparison with fetal bovine serum and considerations on the production protocol
Source: Stem Cell Res Ther. 2022 Apr 4;13:142. doi: 10.1186/s13287-022-02815-1 (PMC8981660; doi:10.1186/s13287-022-02815-1)
Supplement: Supplementary file 3 — Additional file 3. Risk of bias across the studies. [file 13287_2022_2815_MOESM3_ESM.docx]

**Supplementary Table 2.** Risk of bias across the studies

| **Reference** | **Category 1: sample size and processing** | | | **Category 2: suitability of the detection assay** | **Category 3: reproducibility and consistency of described methods** | | **Category 4: completeness and statistical analysis of the results** | |
| --- | --- | --- | --- | --- | --- | --- | --- | --- |
|  | Were at least 3 donors evaluated? | Were all the samples treated equally regardless the experimental group? | Was the assay appropriate for the detection of the specific feature? | | Were all the methods described exhaustively? | Was the experimental setting rigorous? | Were all the results corresponding to each method described? | Were the results reported objectively? |
| Shanbhag, 2020 (11) | Y | Y | Y | | Y | Y | Y | Y |
| Fuoco, 2020 (12) | N (2 donors) | Y | N (MTT for proliferation) | | Y | Y | N (missing FACS result of CD45) | Y |
| Palombella, 2020 (5) | Y | Y | N (MTS for proliferation) | | Y | Y | Y | N (raw data for proliferation) |
| Gao,  2019 (13) | Y | Y | Y | | N (missing protocols for FACS, retrotranscription, RT-PCR, IF, von Kossa staining, proliferation) | Y | Y | Y |
| Becherucci,  2018 (14) | Y | Y | Y | | Y | Y | Y | Y |
| Boraldi, 2017 (15) | N (1 donor) | Y | Y | | Y | Y | Y | Y |
| Pierce, 2017 (16) | N (1 donor) | Y | Y | | N (missing FACS CD35, CD14, CD19) | Y | N (missing FACS result of CD44) | Y |
| Fernandez-Rebollo, 2017 (17) | Y | Y | Y | | N (missing a lot of information) | N (ACT used to normalize gene expression) | Y | N |
| Frese,  2016 (18) | Y | Y | Y | | N (missing information on collagenase solution and adipose differentiation details) | N (not specific adipose differentiation period) | N (missing negative expression of FACS markers) | Y |
| Juhl,  2016 (19) | Y | Y | Y | | Y | Y | N (missing FACS results of CD166 and CD29) | N (missing error bars for PD, no objective data for chromosic aberrations) |
| Riis,  2016 (20) | Y | Y | Y | | N (missing information on collagenase solution) | Y | Y | N (data reported versus an internal control group) |
| Castrèn,  2015 (21) | Y | Y | Y | | N (missing FACS CD90) | N (ACT used to normalize gene expression) | Y | N (proliferation data not clear) |
| Hildner,  2015 (22) | Y | Y | Y | | N (missing information on collagenase solution) | N (only one gene for gene expression) | Y | Y |
| Muraglia,  2015 (23) | Y | Y | Y | | Y | Y | Y | Y |
| Castiglia,  2014 (24) | Y | Y | Y | | N (missing information on FACS antibodies | N (only one gene for gene expression) | Y | N (not clear Chondrogenic differentiation pictures |
| Fekete,  2014 (25) | Y | Y | N (MTS for proliferation) | | Y | Y | Y | N (data reported versus an internal control group, differentiation pictures not clear) |
| Bernardi,  2013 (26) | Y | Y | Y | | N (missing information on FACS antibodies, lymphocyte isolation | Y | N (missing values of FACS, results on karyotype) | Y |
| Kinzebach,  2013 (27) | Y | Y | Y | | N (no description of immunosuppressive experiments, FACS, ELISA) | Y | N (missing results regarding BMSC differentiation and FACS | N (immunosuppressive and FACS results not clear) |
| Menard,  2013 (28) | U | N (different antibiotics concentration) | Y | | N (missing inhibition of immune cell system proliferation) | Y | N (missing qPCR data) | N (FACS data expressed as MFI) |
| Trojahn Kølle,  2013 (29) | Y | N (cell counting at different days) | Y | | Y | N | N (missing data about osteogenesis and Chondrogenesis, chromosal stability, FACS) | N (FACS data and differentiation unclear, general quality of data low) |
| Warnke,  2013 (30) | N (1 donor) | Y | Y | | N (FACS protocol) | Y | N (missing negative results) | N (adipogenesis not clear, low proliferation data) |
| Azouna,  2012 (31) | Y | N (DT calculated at different time points) | Y | | N (retrotranscription not clear, missing CD73 and CD49e with FACS, unclear CFU assay) | N (chondrogenesis with a different medium, protein expression normalized on actin, GAPDH for the gene expression unstable) | N (missing data on FACS for CD31 and CD46 and RANTES with ELISA) | N (gene expression expressed as semi-quantitative data, data of western blot and CFU assay not clear, invaluable pictures, FACS data not clear) |
| Gottipamula,  2012 (32) | Y | Y | Y | | N (missing staining for differentiation, primer for gene expression, lymphocyte isolation and immunosuppressive experiments) | N (gene expression normalized on 18s rRNA) | N (missing statistical analysis) | N (FACS data not clear, gene expression without legend) |
| Cholewa,  2011 (33) | Y | N (evaluation at different cell passages) | N (proliferation with MTT) | | N (not describing staining for differentiation) | N (gene expression normalized with one gene, passaging of cells at 50%, CFU with at least 5 cells) | Y | N (proliferation expressed as raw data, gene expression data not clear, senescence not clear, FACS without quantification) |
| Flemming,  2011 (34) | Y | Y | Y | | N (missing PBMC isolation, CD45 for FACS | Y | N (missing FACS of CD4 and CD80 | Y |
| Castegnaro,  2011 (35) | Y | Y | Y | | N (missing information on FACS antibodies) | Y | Y | N (gene expression as relative quantification) |
| Chevallier,  2010 (36) | Y | Y | Y | | N (missing information on FACS antibodies) | N (gene expression normalized only with one gene) | Y | Y |
| Horn,  2010 (37) | unclear | y | N (MTT for proliferation) | | N (missing CD73 for FACS) | Y | N (missing FACS data) | N (missing pictures of control differentiation) |
| Schallmoser,  2010 (38) | Y | Y | Y | | N (missing information of FACS antibodies) | N (gene expression normalized with one gene) | N (missing data of differentiation and CFU assay) | N (PD not clear) |
| Bieback,  2009 (39) | Y | Y | Y | | N (differentiation not described, FACS, isolation of PBMC) | Y | N (not shown data of telomerase activity) | N (FACS expressed as MFI, secretome expressed as signal intensity) |
| Blande,  2009 (40) | Y | N (differentiation evaluated with different amount of HPL, different number of donors) | N (DT calculated with linear regression on fixed cells) | | Y | Y | Y | Y |
| Prins,  2009 (41) | Y | Y | Y | | N (missing FACS methods for CD235a) | Y | N (missing qualitative FACS data) | N (differentiation pictures not clear) |
| Capelli,  2007 (42) | Y | Y | Y | | N (missing information on FACS antibodies, PBMC isolation) | Y | Y | Y |
| Schallmoser,  2007 (43) | Y | Y | Y | | N (missing differentiation and staining protocols, description of FACS antibodies, CFU assay) | Y | Y | N (CFU results not clear, most results are only descriptive, FACS expressed as MFI) |
| Doucet, 2005 (3) | Y | Y | Y | | N (missing details of HPL preparation, ALP detection and activity, lymphocyte isolation, proliferation method) | Y | Y | N (FACS reported as arbitrary units) |

Y = yes; N = no; U = unclear
