# Supplementary material for: Systematic review and meta-analysis on the use of human platelet lysate for mesenchymal stem cell cultures: comparison with fetal bovine serum and considerations on the production protocol
Source: Stem Cell Res Ther. 2022 Apr 4;13:142. doi: 10.1186/s13287-022-02815-1 (PMC8981660; doi:10.1186/s13287-022-02815-1)
Supplement: Supplementary file 4 — Additional file 4. Sensitivity Analysis—Forest plots of doubling time for FBS 10% versus HPL 10% and FBS10% versus HPL 5%. [file 13287_2022_2815_MOESM4_ESM.docx]

**Sensitivity analysis**

**
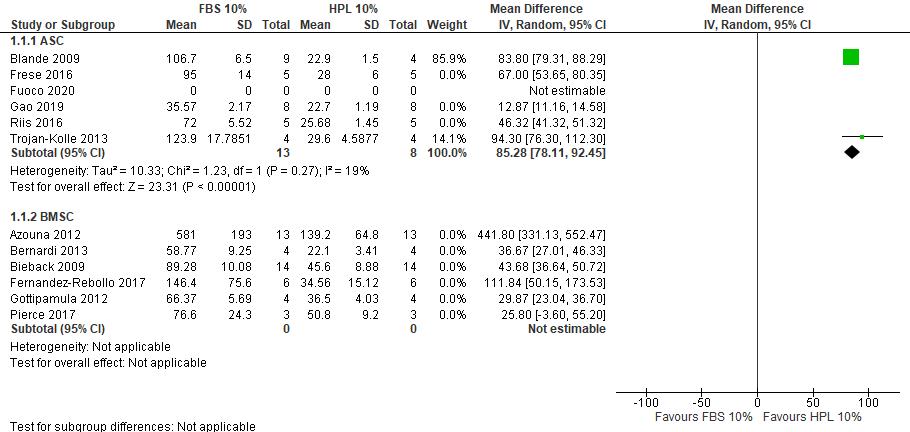
**

**Figure S1**. Sensitivity analysis- Forest plot of doubling time for FBS 10% versus HPL 10%. For both BMSCs and ASCs the DT decreased with 10% HPL compared to 10% FBS

**
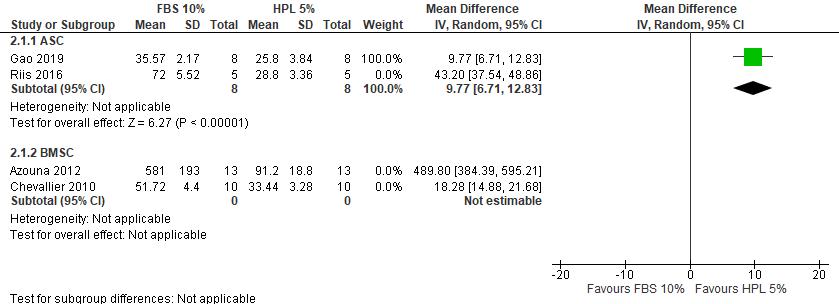
**

**Figure S2.** Sensitivity analysis - Forest plot of doubling time for FBS 10% versus HPL 5%. The supplementation with 5% HPL is slightly favored compared to 10% FBS
